# Dynamic Circadian Protein–Protein Interaction Networks Predict Temporal Organization of Cellular Functions

**DOI:** 10.1371/journal.pgen.1003398

**Published:** 2013-03-28

**Authors:** Thomas Wallach, Katja Schellenberg, Bert Maier, Ravi Kiran Reddy Kalathur, Pablo Porras, Erich E. Wanker, Matthias E. Futschik, Achim Kramer

**Affiliations:** 1 Laboratory of Chronobiology, Charité–Universitätsmedizin, Berlin, Germany; 2 Sysbiolab, University of Algarve, Faro, Portugal; 3 Max Delbrück Center for Molecular Medicine, Berlin, Germany; University of Pennsylvania, United States of America

## Abstract

Essentially all biological processes depend on protein–protein interactions (PPIs). Timing of such interactions is crucial for regulatory function. Although circadian (∼24-hour) clocks constitute fundamental cellular timing mechanisms regulating important physiological processes, PPI dynamics on this timescale are largely unknown. Here, we identified 109 novel PPIs among circadian clock proteins via a yeast-two-hybrid approach. Among them, the interaction of protein phosphatase 1 and CLOCK/BMAL1 was found to result in BMAL1 destabilization. We constructed a dynamic circadian PPI network predicting the PPI timing using circadian expression data. Systematic circadian phenotyping (RNAi and overexpression) suggests a crucial role for components involved in *dynamic* interactions. Systems analysis of a global dynamic network in liver revealed that interacting proteins are expressed at similar times likely to restrict regulatory interactions to specific phases. Moreover, we predict that circadian PPIs dynamically connect many important cellular processes (signal transduction, cell cycle, etc.) contributing to temporal organization of cellular physiology in an unprecedented manner.

## Author Summary

Circadian clocks are endogenous oscillators that drive daily rhythms in physiology, metabolism, and behavior. In mammals, circadian rhythms are generated within nearly every cell; and, although dysfunction of circadian clocks is associated with various diseases (including diabetes and cancer), the molecular mechanisms linking the clock machinery with output pathways are little understood. Since essentially all biological processes depend on protein–protein interactions, we investigated here on a systems-wide level how time-of-day-specific protein–protein interactions contribute to the temporal organization of cellular physiology. We constructed a circadian interactome using experimentally generated protein–protein interaction data and made this network dynamic by the incorporation of time-of-day-dependent expression data. Interestingly, systematic genetic network perturbation (RNAi and overexpression) suggests a crucial role for circadian components involved in *dynamic* interactions. Systems analysis of a global network revealed that interacting proteins are in the liver significantly more expressed at similar daytimes likely to restrict regulatory interactions to specific circadian phases within cells. Overall, circadian protein–protein interactions are predicted to dynamically connect important cellular processes (signal transduction, cell cycle, etc.) using—very often—protein modules with components co-expressed in time, shedding new light on the daily organization of cellular physiology.

## Introduction

Circadian clocks are endogenous oscillators conserved in nearly all living organisms that drive ∼24 hour cycles in physiology and behavior. In mammals, the circadian oscillator is composed of interconnected transcriptional translational negative and positive feedback-loops which generate circadian rhythms at the molecular level. Within this gene-regulatory network, a precise timing of gene expression, protein–protein interactions (PPIs) as well as posttranscriptional and posttranslational modifications is essential for sustaining circadian rhythms with normal dynamics [1]–[3]. The interaction between the transcription factors CLOCK and BMAL1, which has been discovered in a yeast-two-hybrid (Y2H) screen [4], is crucial for the activation of the *Period* (*Per1*, *Per2*, *Per3*) and *Cryptochrome* (*Cry1*, *Cry2*) genes. PER and CRY proteins form large complexes that inhibit their own transcription by binding directly to the CLOCK/BMAL1 complex during the late night [5].

Circadian rhythms in gene expression are pervasive – 2–10% of the transcriptome in a given tissue is under circadian control [6], [7]. Consequently, also a large fraction of the proteome is thought to be regulated in a time-of-day dependent manner, although systems-wide studies of circadian protein abundance rhythms are still rare (however, see [8]). Cellular functions are increasingly recognized to be regulated by protein complexes or ‘modules’ [9], thus PPIs and their timing are predicted to be crucial. In most cases, in which PPIs exert a regulatory function, such interactions are transient and occur only under specific conditions, *e.g*. as a response to a signal, after binding of a co-factor or when the expression of one or both partners is induced in response to a changing cellular condition. Circadian clock regulation of cellular functions *via* PPIs can be accomplished by restricting important interactions to specific times of the day. In the circadian oscillator, many of the known PPIs also happen predominantly at specific times of the day, *e.g*. PER/CRY complexes bind to CLOCK/BMAL1 in the late night to inhibit transactivation [5]. Here, the temporal binding profile correlates with the abundance profiles of PER and CRY proteins. While these examples demonstrate the fundamental importance of precisely timed PPIs for the circadian clockwork, we are still far from a comprehensive view of the PPI network among circadian oscillator proteins and their dynamics. Furthermore, the extent of a regulation of circadian output processes *via* time-of-day dependent PPIs is largely unknown.

To elucidate unknown regulatory mechanisms within the circadian clockwork we have systematically mapped PPIs among 46 circadian components using high-throughput Y2H interaction experiments. We have identified 109 so far uncharacterized interactions and have successfully validated a sub-fraction *via* co-immunoprecipitation experiments in human cells. Among the novel PPIs we have identified modulators of CLOCK/BMLA1 function indicating a role for protein phosphatase 1 (PPP1) in the dynamic regulation of BMAL1 abundance. Furthermore, to generate a more comprehensive circadian PPI network we have enriched and extended our experimental network with additional validated interactions and interaction partners from literature, some of which seem to be essential for normal circadian dynamics. The integration of circadian mRNA expression profiles from mouse liver allowed us to predict the interaction dynamics within our network in hepatocytes. Using systematic genetic perturbation studies (RNAi and overexpression in oscillating cells) we propose a crucial role of dynamic regulation (*via* rhythmic PPIs) for the molecular clockwork. Furthermore, we have extracted a dynamic modular organization as a pervasive circadian network feature possibly contributing to time-of-day dependent control of many cellular processes. Systems analysis on a global scale regarding circadian regulation of biological processes *via* rhythmic PPIs suggests a time-of-day dependent organization of the interactome. Altogether our data should provide a valuable resource of circadian PPIs within hepatocytes that are important not only for keeping the pace of the molecular clockwork but likely also for the control of cellular physiology.

## Results

### Large-Scale Yeast-Two-Hybrid Interaction Analysis with Circadian Clock Components

**Figure 1:**
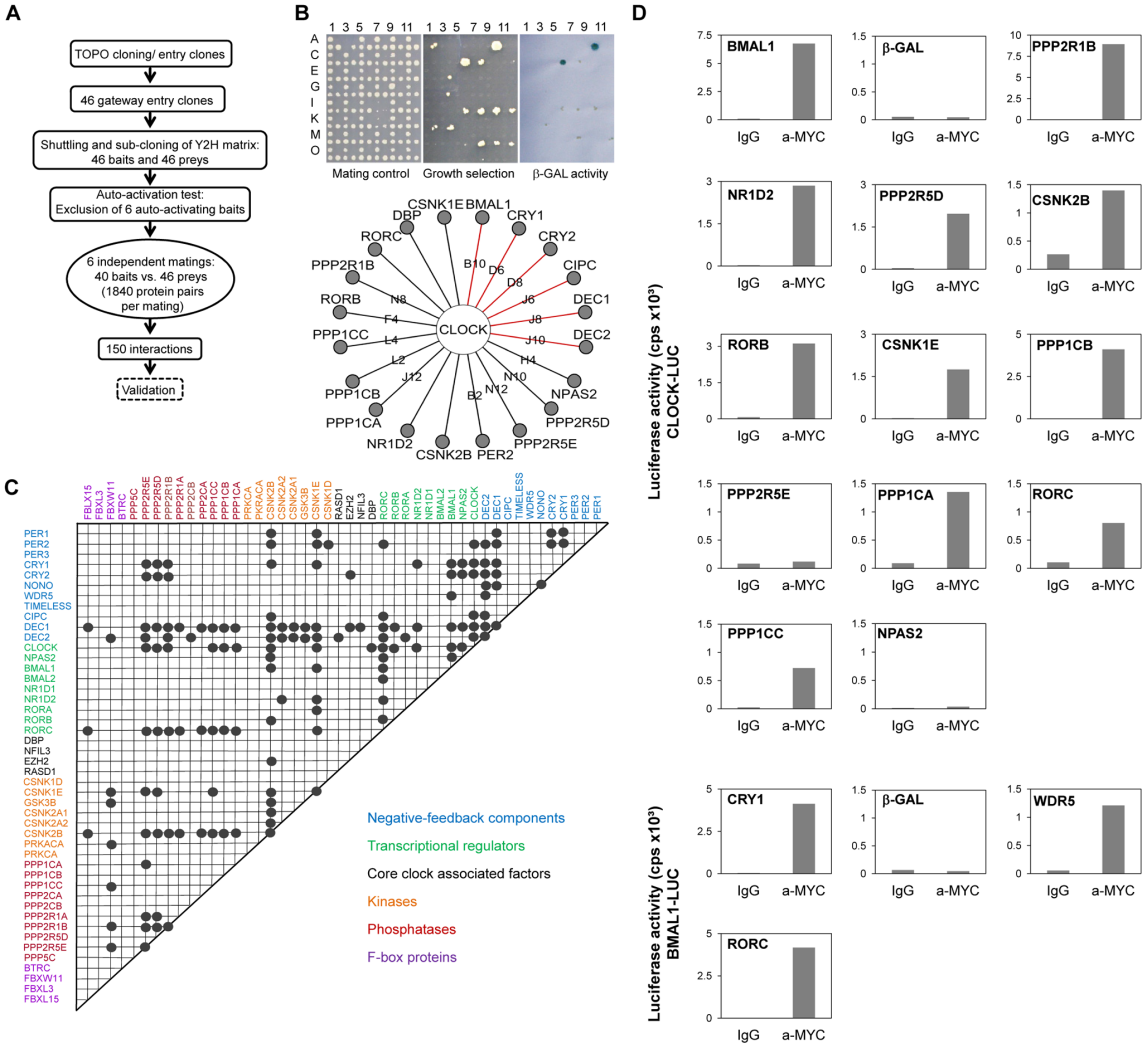
Systematic Interaction Mapping between 46 Circadian Clock Proteins and Associated Components. (A) Matrix based high-throughput yeast-two-hybrid interaction screen. (B) CLOCK interactors: Mating controls (top left); upon PPI reporter genes are activated (top middle: HIS, URA for growth selection, top right: lacZ for β-galactosidase activity). Bottom: Detected interactions with CLOCK; red lines: interactions previously discovered in yeast (see also Figure S1). (C) Clock protein interaction matrix. Circles: interactions between two components not differentiating between bait and prey configuration. (D) Validation of new CLOCK and BMAL1 interactions in mammalian cells. HEK293 cells expressing CLOCK- or BMAL1-luciferase fusions were transfected with MYC-tagged components. Luciferase activity in anti-MYC co-immunoprecipitates is presented for one representative result of at least two independent experiments with similar results (for method and input controls also see Figure S2). MYC-β-galcactosidase fusions served as negative, MYC-BMAL1 and MYC-CRY1 as positive controls, respectively.

To systematically map the PPIs within the circadian clock regulatory network, we performed a matrix-based two-hybrid screen in yeast with 46 known or assumed clock or clock-associated components (Figure 1A; for justification of our selection see Text S1 and Table S1). In this screen, each potential interaction was tested individually in six replicas to increase screening saturation thereby minimizing the number of false negatives (for details on the method, see Figure S1 and [10]). After excluding transcriptionally autoactive components, we performed 11,040 individual yeast-two-hybrid experiments monitoring growth on selective medium and β-galactosidase activity as readouts for interaction (Figure 1B). Thereby, we identified 150 interacting protein pairs that occurred at least in two independent experiments (Figure 1C). We could reproduce a large number (41 of 104) of previously described interactions (*e.g.*, CLOCK-BMAL1, PERs-CRYs, CRYs-BMAL1; see Figure S2A and Table S1) corresponding to a rather high sensitivity (of ∼40%) for a yeast-two-hybrid assay, which is usually only about 25% [11]. Importantly, among the 150 detected PPIs we found 109 previously unknown PPIs between circadian clock proteins. For example, we detected interactions between DEC1/2 and CRY1/2, between CLOCK and RORβ/γ, between CLOCK and the α-catalytic subunit of protein phosphatase 1 (PPP1Cα) as well as between BMAL1 and WDR5 (Figure 1B, 1C).

To test whether the PPIs discovered in yeast can also occur in mammalian cells, we performed co-immunoprecipitation experiments in HEK293 cells. As representatives for the novel PPIs we focused on the interactions of the transcriptional activators CLOCK and BMAL1 – central players within the circadian clock gene-regulatory network, whose functional modulation by interacting proteins is likely to be highly relevant for normal circadian rhythms. Twelve of the 14 (*i.e*., 86%) novel CLOCK and BMAL1 interactions found in yeast were validated using co-immunoprecipitation (Figure 1D and Figure S2), suggesting that a substantial proportion of all interactions identified in yeast can also take place in mammalian cells.

### Enrichment, Extension, and Topology of the Circadian PPI Network

**Figure 2:**
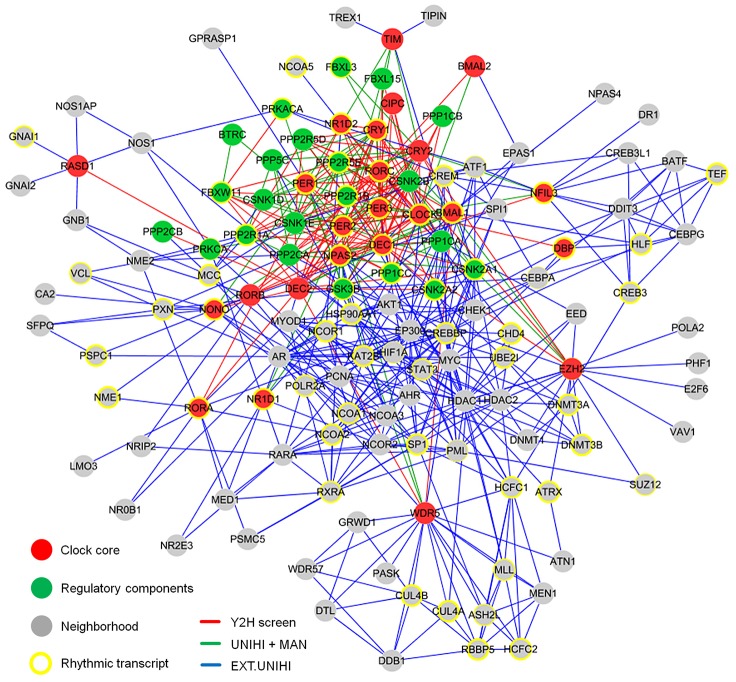
The Circadian Protein–Protein Interaction Network. The circadian interaction network integrates different interaction sources and visualizes 134 proteins with 625 interactions. Red lines: interactions discovered in yeast (see Figure 1); green lines: previously described (and detected in our Y2H screen) interactions (source: UniHI database and/or literature (MAN)); blue lines: interactions in network extension (EXT - stored in UniHI), i.e. between clock core and regulatory components and neighborhood components (see also Figure S3A). Yellow border: components with a rhythmic transcript in mouse liver [6]. Border width: significance for rhythmic expression.

To understand the structure and the organizing principles of the complex web of interactions occurring between circadian clock components, we created an interaction network using our novel yeast-two-hybrid interaction data together with previously published interactions among these components. This is necessary, since the sensitivity of any high-throughput PPI detection assay is limited [11] and thus the false-negative rate is expected to be rather high. In addition, we extended this network by adding known interacting proteins (direct ‘neighbors’) of our network components (except regulatory components such as kinases, phosphatases and F-box proteins, which are known to be involved in many other cellular processes) to get an idea how the circadian PPI network is embedded in the cellular interactome (Figure S3A). To this end, we used PPI data extracted by human experts from literature and stored in the UniHI database [12], however only those, for which experimental validation exists. We did not use predicted PPIs based on orthology or from computational text mining. Thereby, a large PPI network with 134 components and 625 PPIs was created consisting of a circadian clock core (24 components), regulatory components (22 components) and the neighborhood (88 components; Figure 2 and Table S1).

For this network, a mean shortest path length between any two proteins of 2.8 links was calculated, *i.e.* most proteins are very closely linked to each other indicating a ‘small world’ type of network [13]. Like many other PPI networks [14], the circadian network has properties of a ‘scale-free’ network, *i.e.* many proteins have few and few proteins have many interactions (Figure S3B). On average each component has 8.4 interaction partners, however, 11 proteins are highly connected with more than 20 interactions (*e.g.* CLOCK, BMAL1, PER2, CREBBP, DEC1, AR, HDAC1). Network topology analysis further revealed that our network is hierarchically organized, *i.e.* highly connected components (so-called ‘hubs’) link network regions with less connected components, which themselves tend to form clusters (Figure 2; Figure S3B).

### Characterization of the Circadian Clock Network Neighborhood

**Figure 3:**
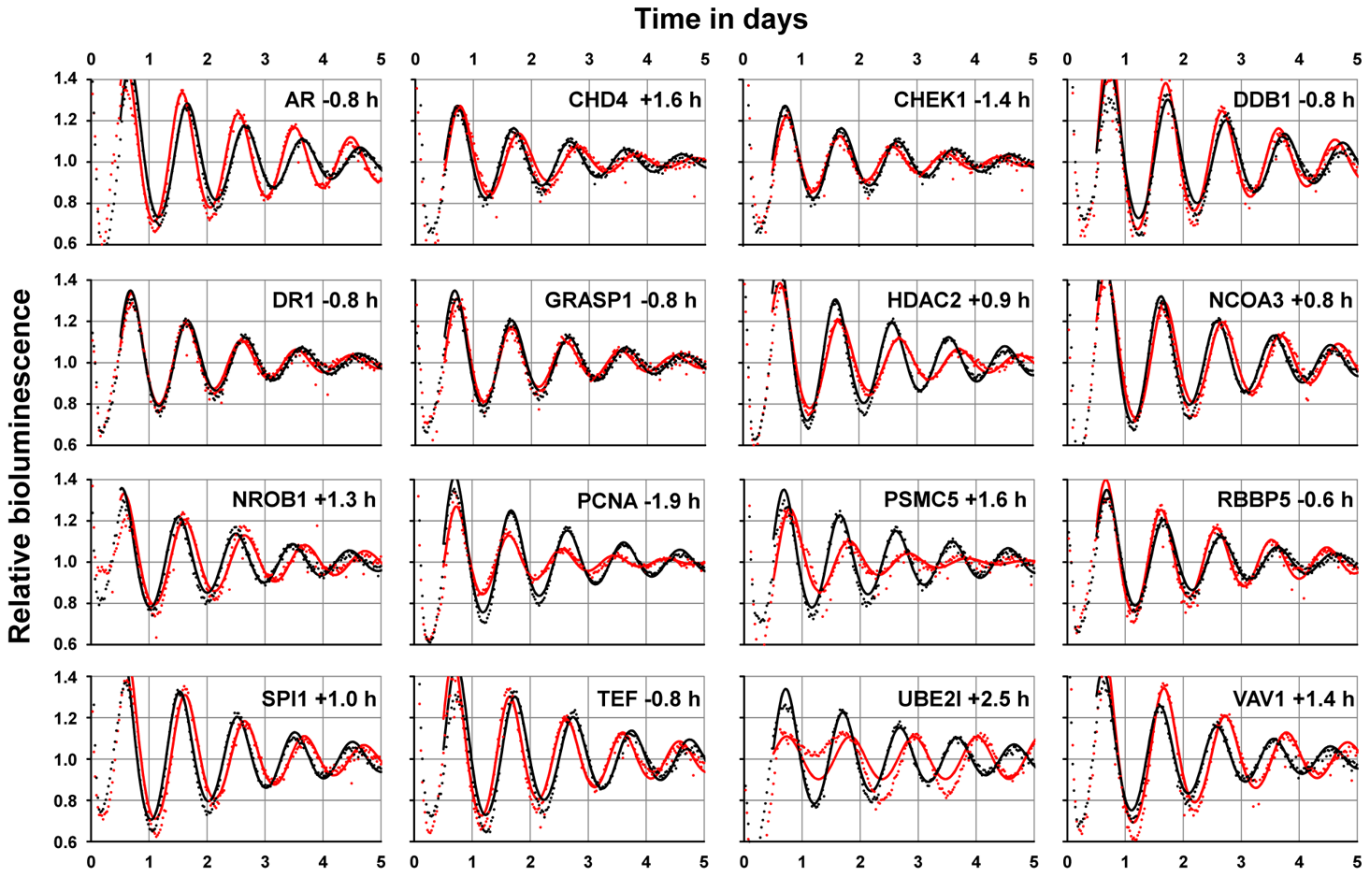
Network Neighborhood Contains Clock Modulating Components. Systematic RNAi-mediated downregulation of network neighborhood genes in dexamethasone-synchronized U2OS cells harboring a *Bmal1*-promoter luciferase reporter. Shown are altered oscillation dynamics (red dots with corresponding fit lines) for 16 genes achieved by individual RNAi constructs (see Table S2). For twelve genes, two RNAi constructs resulted in similar phenotypes, for nine genes only one construct was available in our laboratory library. Black dots with corresponding fit lines are controls representing the mean values of at least 80 irrelevant constructs. Period deviations from controls are shown.

Proteins in the direct network neighborhood that interact with circadian clock core components might be relevant for regulating clock output functions, but could also include yet unknown proteins important for modulating the circadian clock machinery; *i.e.* they might be clock components themselves. To test the latter possibility, the expression of 88 neighborhood genes was systematically downregulated by RNAi in human U2OS cells. These cells possess robust circadian rhythms in cell culture, and RNAi-mediated downregulation of canonical clock genes has been shown to copy circadian phenotypes of classical knockout mice [15], [16]. We monitored circadian rhythms *via* a stably integrated *Bmal1* promoter-luciferase reporter construct and identified 21 components of the neighborhood that altered circadian period upon knockdown by at least 0.5 hours (Figure 3 and Table S2). For example, downregulating the cell-cycle kinase CHEK1 (that can interact with TIMELESS and CK2) significantly shortened the circadian period by more than 1 hour, while downregulating the DNA helicase binding protein CDH4 (that is reported to interact with RORγ) lengthened it. In addition, knocking down the androgen receptor (AR), which interestingly was found to interact with many proteins (including NONO, GSK3β, HDAC1, CREBBP, UBE2I and NCOR1/2), results also in a shortening of the circadian period by almost one hour. Although these results need additional in-depth validation, the relatively high number of clock modulating components in the network neighborhood suggests the presence of yet uncharacterized mechanisms in the molecular circadian oscillator, as suggested earlier [15], [16]. Future studies are needed to investigate whether these circadian phenotypes in U2OS cells are similar in other cell types and *in vivo*.

In addition to possibly being novel clock components, proteins in the network neighborhood might also connect specific cellular processes to circadian control by means of directly interacting with clock components. Such interactions are likely time-of-day dependent, which may be accomplished by rhythmic abundance levels of one or both of the interaction partners. Therefore, we hypothesized that the whole network but also the neighborhood alone are significantly enriched in components with rhythmic abundance levels. This is indeed the case – at least if we consider (due to the lack of protein abundance data) mRNA expression profiles of network components in mouse liver tissue – the circadian transcriptome with highest available temporal (1 h) resolution [6]. Of the 134 network components, 65 (49%) show a significantly rhythmic mRNA expression profile in liver, a highly significant enrichment when compared to a random selection of genes from this expression data set (p<10^−6^; Chi-squared test). This may not be surprising, since the network as a whole contains circadian oscillator components, many of which are known to be rhythmically transcribed. However, if we analyze the neighborhood separately, we still find a significant enrichment (p<10^−4^; Chi-squared test) in components that are rhythmically transcribed: of the 88 components in the neighborhood, 38 (43%) are rhythmically expressed in the liver (Figure 2, yellow circles) suggesting that PPIs in the hepatocyte circadian clock network might indeed be a means to mediate rhythmic control of cellular physiology.

### A Dynamic Circadian PPI Network

**Figure 4:**
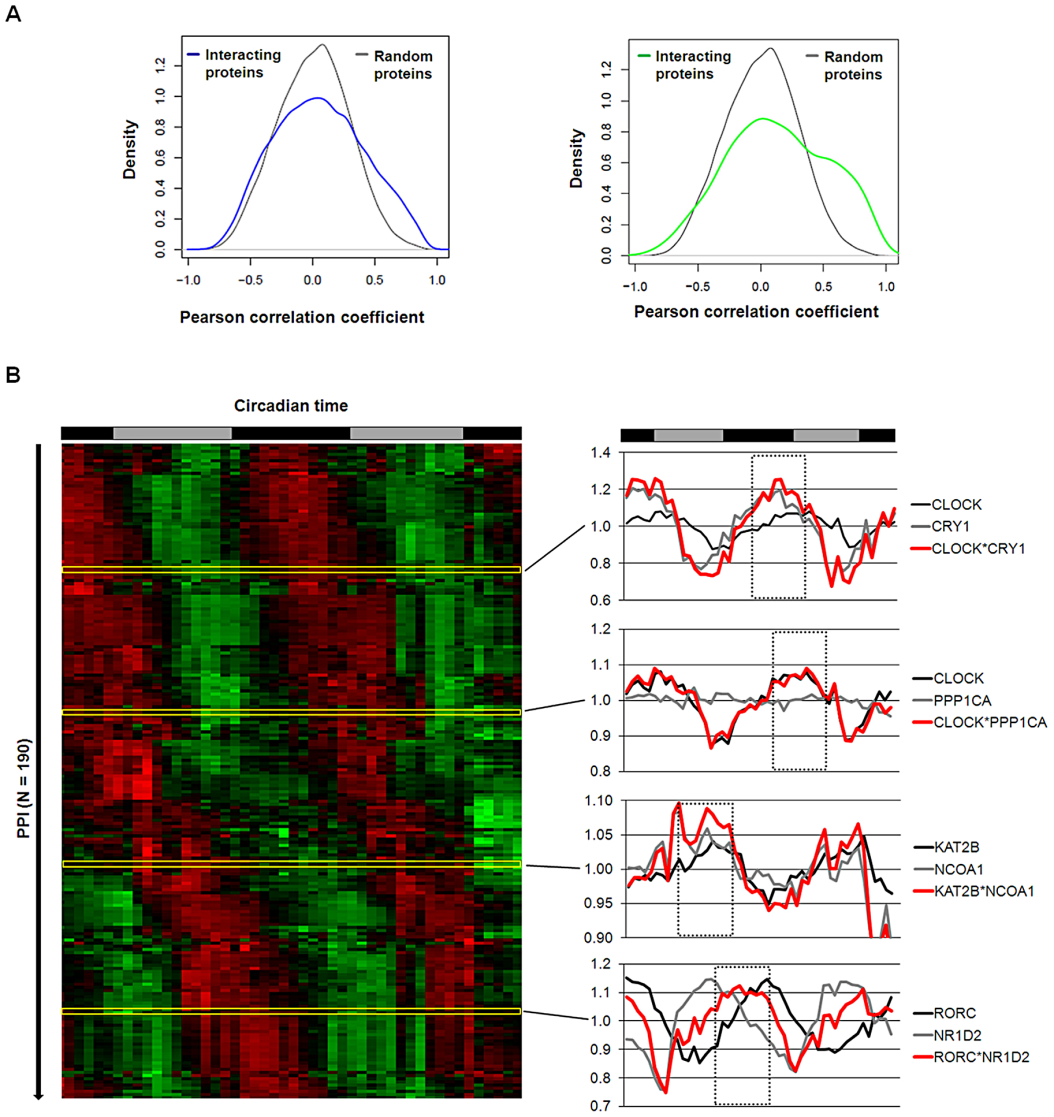
Interaction Dynamics within the Liver Circadian Protein–Protein Network. (A) Interacting proteins are more likely to be co-expressed in time. Left: Co-expression of interacting proteins was calculated using the Pearson correlation coefficient (PCC) of circadian expression profiles in liver [6] and compared to randomly selected protein pairs. Among interacting proteins co-expressed proteins (i.e. PCC>0.5) are significantly overrepresented (Chi squared test: p<10^−15^). 13% of interacting proteins have a PCC>0.5 compared to 4% for random pairs. Right: analogous analysis for the circadian PPI network. Co-expressed (PCC>0.5) interacting proteins are highly overrepresented (Chi squared test: p<10^−15^; 22% compared to 4% with PCC>0.5). (B) Left: heat map representing the predicted dynamics of protein–protein interaction based on their liver expression profiles. Interactions were classified as rhythmic if the product of their expression vectors shows highly significant periodic expression (FDR<10^−5^). Right: examples for interaction pairs and their predicted interaction phase. Red lines: products of individual transcript profiles of two interacting proteins. Dotted rectangles highlight predicted phase of interaction.

At what time of day do the PPIs in the circadian network occur or – in other words - can we predict dynamic properties of our (still static) network? Again, hypothesizing that a PPI more likely happens at times, when the interaction partners are co-expressed, we again used transcriptome data (from mouse liver) [6] as a validated proxy for protein abundance [17] – an approach successfully used also for the yeast interactome [18]. To first test this hypothesis for PPIs in general (*i.e.* beyond our circadian network), we compared the Pearson correlation coefficient (PCC) of transcript levels (as a measure for co-expression) for all pairs of interacting liver proteins present in the UniHI interactome database and for which we have time-resolved expression data [6] with the PCC of randomly chosen pairs. Interestingly, we found that interacting liver proteins are significantly more likely to be expressed at similar circadian times (PCC>0.5 with PCC ranging from −1 to 1; p<10^−15^ Chi-squared test; Figure 4A left and Figure S5). These data suggest that circadian co-expression may be a common feature to restrict regulatory interactions to specific times of the day. This assumption is supported by the fact that liver proteins with many interaction partners – which are known to exert regulatory functions - are more likely to be rhythmically expressed (p<10^−10^; Wilcoxon Rank test) and *vice versa*, *i.e.* proteins with rhythmic transcripts have statistically more interaction partners than constitutively expressed proteins (p<10^−5^; Chi-squared test). Interestingly, also the circadian PPI network displays these properties: interaction between proteins is more likely, when both proteins are co-expressed in time (Figure 4A, right).

Based on our results above, we hypothesized that many PPIs happen at specific times of the day. Therefore, we assigned to each PPI in our network a circadian phase, at which the corresponding components are predicted to interact in the liver based on their transcript expression. To this end, we approximated the abundance of the complex of two proteins as the product of their expression profiles. Derived time series for the interaction complexes were subsequently examined for 24 hour periodicity with a stringent threshold (false discovery rate FDR<10^−5^) resulting in the prediction of a dynamic circadian PPI network with 193 individual protein pairs interacting at specific circadian phases (Figure 4B and Table S3). Interestingly, PPIs in the liver seem to be distributed over the whole circadian cycle. Beyond the dynamic interactions that occur among circadian core components in this network, we extract many time-of-day specific putative regulatory interactions within the neighborhood. For example, the lysine acetyltransferase KAT2B is predicted to bind to the nuclear receptor coactivator NCOA1 - two proteins involved in transcriptional regulation - during the late day, which may hint to a time-of-day specific function of these proteins. Nevertheless, it should be noted that this prediction is only valid for the liver, since the identity of rhythmic transcript is highly tissue-specific [19]. In addition, we are aware that the restriction to transcript (and not protein) profiles, the possible tissue-specificity of certain PPIs and also a potential competitive nature of the possible interactions pose limitations to this analysis (but see below for experimental validation of the daytime dependent interaction between PPP1Cα and CLOCK/BMAL1). However, such a framework offers the possibility to globally analyze processes controlled by circadian PPIs in a time-specific manner.

### Role of Dynamic Interactions for the Circadian Oscillator

Network components with many interaction partners - so-called ‘hubs’- not only have important organizing properties in scale-free networks; they are also (controversially) discussed to be more essential for life (at least in yeast, *Drosophila* and *C. elegans*; [20]). In a dynamic network, two types of hubs have been proposed – ‘party hubs’, which interact with their partners predominantly at similar times, and ‘date hubs’, whose interactions mostly occur at different times or locations [18]. In yeast, especially the ‘date hubs’ are described to be global regulators for the cellular physiology suggesting a prominent role of dynamic regulation within complex networks.

**Figure 5:**
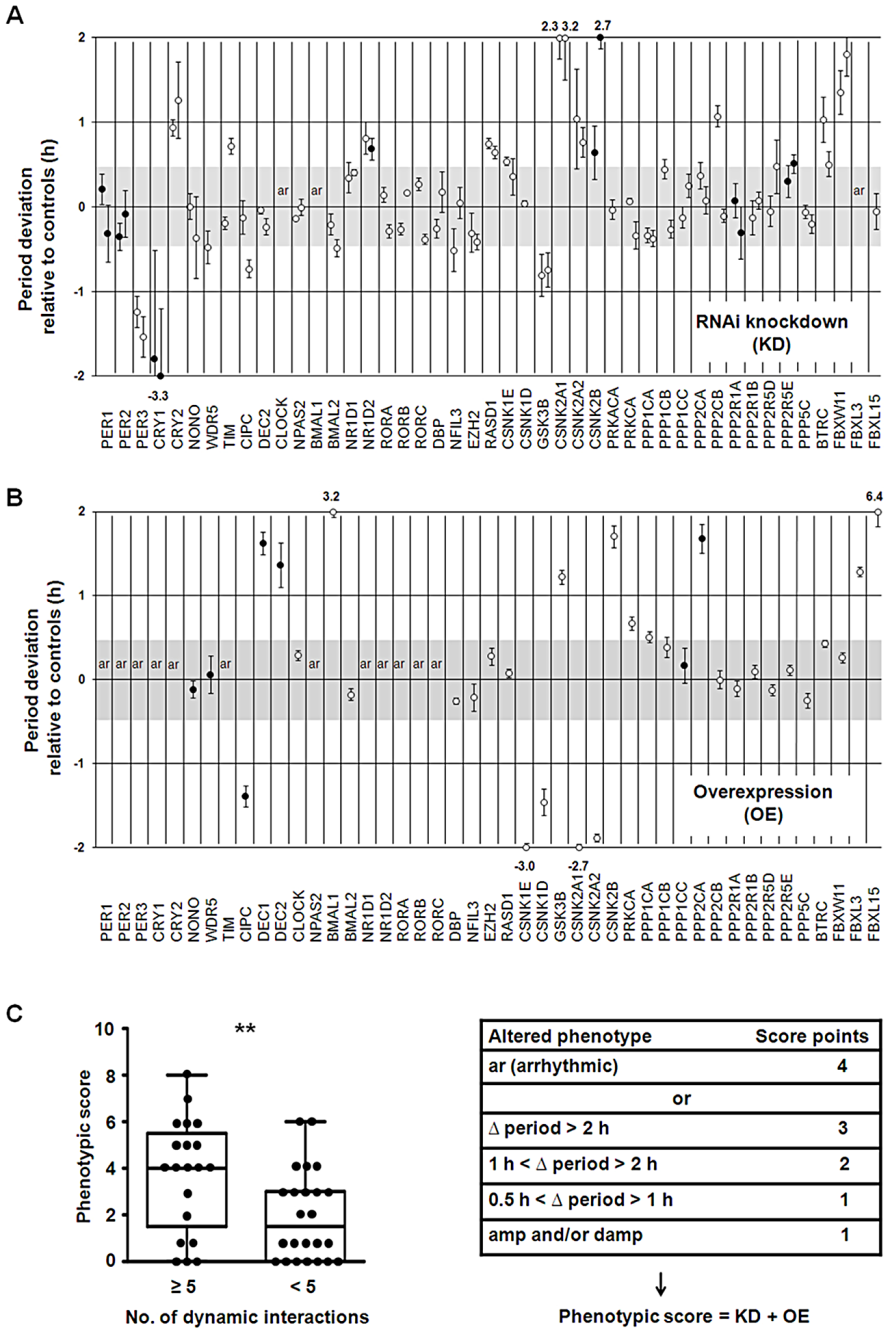
Importance of Dynamic Interactions for Circadian Rhythmicity. (A) Systematic RNAi-mediated silencing of circadian clock core and regulatory components. RNAi constructs were lentivirally delivered into U2OS cells harboring a *Bmal1*-promoter luciferase reporter and oscillation dynamics were monitored for several days (see also Figure 3). Circles represent the difference in period (± s.e.m.; n = 3 independent experiments) relative to non-silencing controls (n>10) for two RNAi constructs (if available). Filled circles show additional amplitude and/or damping phenotypes. Cells were classified as arrhythmic (ar) if the fit to a cosine function resulted in a low correlation coefficient (see Text S1). Period deviations of more than 2 hours are given (see also Figure S4A). (B) Systematic overexpression of circadian clock core and regulatory components. Experiments were performed with lentivirally delivered overexpression constructs as described in (A) (see also Text S1). Results of three independent experiments (± s.e.m.) are given (also see Figure S4B). (C) Correlation of circadian phenotype with number of dynamic interactions. The combined phenotypic score from silencing and overexpression experiments is significantly different for components with many dynamic interactions (≥5) compared to those with few (<5) (t-test: ** p<0.01; Mann Whitney test: ** p<0.01).

To test, whether in our network ‘hub’ proteins are essential for the trait ‘circadian rhythmicity’ - *i.e.* for generating and maintaining circadian rhythms – we correlated circadian phenotypes obtained upon genetic perturbation (see below) with topological characteristics of network components. For perturbing the network experimentally and assigning an essentiality score (for circadian rhythmicity) to each component, we (i) systematically knocked down and (ii) overexpressed every component of the core and the regulatory part (not the neighborhood) of the network. We performed these experiments in human U2OS reporter cells (as described above) and analyzed the effect on circadian dynamics. While we could reproduce most of the phenotypes that have been known from studies with classical knockout models (*e.g.* the opposite period phenotypes upon *Cry1* and *Cry2* deletion as well as arrhythmicity upon *Bmal1* and *Clock* knockout), we detected interesting novel phenotypes such as period lengthening for *Rev-Erbβ* (*Nr1d2*) downregulation (Figure 5A and Figure S4A). As examples for phenotypes detected upon clock protein overexpression, *Dec1* or *Dec2* as well as *Fbxl15* (the homologue to *Drosophila Jetlag*) led to a substantial period lengthening (∼1.5 hours and ∼6 hours, respectively) (Figure 5B and Figure S4B). For each network component tested we combined the downregulation and overexpression phenotypes in a ‘phenotypic score’ (for rules, see Text S1) to be able to correlate it with network properties of the individual components. Surprisingly, we did not see a correlation of phenotypic score with the number of interactions as it has been observed in more global networks of yeast, *Drosophila* and *C. elegans* [20]. In other words, ‘hub’ proteins apparently are not more important for circadian rhythmicity than components with a lower connectivity. However, proteins that are predicted to be involved in *dynamic* interactions (at least in liver) turned out to be more essential for circadian rhythm generation (*t*-test: p<0.01; Mann-Whitney U test: p<0.01; Figure 5C, Table S4). For example, CLOCK, BMAL1, PER3 and CRY1 – to which we assigned 24, 21, 18 and 9 dynamic interaction, respectively – are especially important for circadian dynamics (Figure 5A–5C). Importantly, factors that have a rhythmic transcript *per se* (without taking PPIs into account) are not significantly more likely to be essential for circadian rhythms (independently of whether we set the rhythmicity threshold at a FDR of 0.05 or 0.01; not shown). While we did not test the importance of rhythmic PPIs for circadian dynamics directly, this correlative result suggests that the more rhythmic interactions a protein is involved in, the more important it is for normal circadian rhythmicity. In addition, 40% (10 of 25) of the 45 ‘hubs’ that qualify as ‘party hubs’ in the liver (Table S3) show circadian phenotypes upon genetic perturbation, while the only two ‘date hubs’ (CLOCK and AR; Figure S5) both are sensitive to perturbation - in line with the described prominent role of ‘date hubs’ for network organization [18].

### Regulation of Cellular Processes via Dynamic PPIs

**Figure 6:**
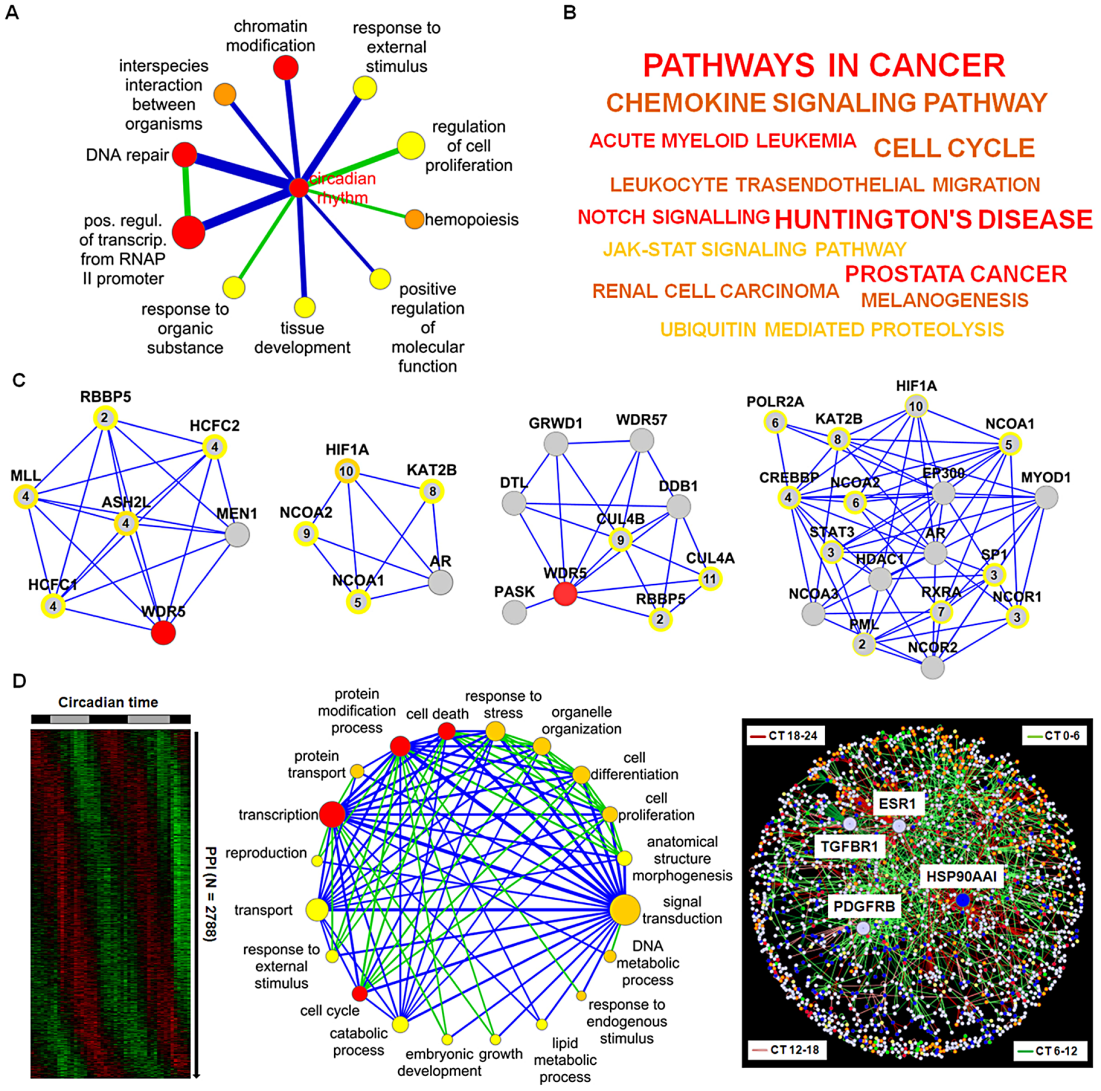
Prediction of Circadian Output Regulation. (A) Coupling of biological processes via predicted dynamic PPIs. Node size: number of genes in GO category (significance (FDR<0.25, <0.01 and <0.0001 from yellow to red). Edge width and color: number of interactions and enrichment in dynamic interactions (blue: p<0.001; green: p<0.1) (for details see main text and Text S1). (B) KEGG pathway analysis of network neighborhood. From yellow to red (p<0.02, <0.0005 and <0.0002). Font: number of components in each category. (C) Highly connected clusters. Modules with histone methyltransferase complex, transcription coactivator activity, response to DNA damage stimulus and histone acetyltranferase activity as significant GO terms (left to right). Peak expression times are given within circles (see Figure S6A for all modules identified). (D) Left: Predicted dynamic PPIs within the liver (see Figure S6B). Middle: coupling of biological processes via predicted dynamic interactions in the liver. Node color: significant dynamic interactions (p<0.25, <0.0001, p<10^−8^ from yellow to red); edge color: enrichment in dynamic interactions (blue: p<10^−16^; green: p<10^−5^). Right: dynamic global PPI network. Node color: most significant biological processes, i.e. cell cycle (green), cell death (red), protein modification (yellow), signal transduction (blue) and transcription (orange).

Are dynamic PPIs also important for the regulation of cellular events? To predict such regulations, we first assigned to each network component one or more specific gene ontology (GO) categories from a reduced, less redundant and more distinct set of GO categories (for details see Text S1). Secondly, using the information whether a PPI is likely to be dynamic or not (see Figure 4B), we investigated which cellular processes (as represented by GO categories) are significantly connected *via* dynamic PPIs (see Text S1). In other words, we tested whether in our network dynamic interactions are over-represented in the total set of interactions between a pair of GO categories. This resulted in a “process network” with 12 dynamic links between 11 biological processes with ‘circadian rhythm’ as the central hub. This hub is rhythmically connected with GO terms such as ‘DNA repair’, ‘transcriptional regulation’ and ‘response to external stimulus’ (Figure 6A; Table S5). A strong association of the circadian clock network with these processes relevant for *e.g.* cancer and cell-cycle is also found by (i) Kyoto Encyclopedia of Genes and Genomes (KEGG) pathway analysis of the network neighborhood only (Figure 6B) and (ii) the significant (p<10^−8^; Chi-squared test) enrichment of the network neighborhood with cancer-associated genes (as reported in the Cancer Gene Census list (www.sanger.ac.uk/genetics/CGP/Census)).

How are these rhythmically regulated processes connected in our network - by individual components or rather by functional modules consisting of interconnected components? We explored our circadian PPI network topology for clusters of highly connected proteins (structural modules) and identified 11 different modules within the circadian network (Figure 6C; Figure S6A and Text S1) often with module components co-expressed in time suggesting that modular organization within the circadian PPI network might contribute to a coherent functional regulation of hepatocyte processes by the circadian clock. This is also supported by a high cluster coefficient (0.38) of the circadian network compared to randomized networks (0.14±0.01) (Figure S3C).

Next, we analyzed whether time-of-day dependent interaction of cellular processes *via* PPIs can also predicted on a more global scale. To this end, we first assigned 2788 rhythmic PPIs (using the approach described above - see Figure 4B) to a global interactome derived from the UniHI database (Figure 6D left) and then searched for GO terms (‘biological process’) that are significantly connected *via* predicted dynamic PPIs (Figure S6B). We extracted a network of 20 biological processes with 89 dynamic links (for details see Text S1). The central ‘hub’ of this ‘process network’ constitutes the term ‘signal transduction’ (Figure 6D middle and Figure S6C) suggesting a possible time-of-day dependent modulation of hepatocyte events such as ‘protein transport’, ‘response to stress’ and ‘cell death’ by signaling pathways *via* rhythmic PPIs.

To characterize the underlying PPI network properties, we constructed a global dynamic interactome and found that it again has ‘scale-free’ properties with 269 dynamic ‘hubs’, *i.e.* proteins with at least 5 predicted dynamic interactions. The protein with the most predicted rhythmic interactions (79 of 105 in total) is heat-shock protein HSP90AA1 – a factor required for proper protein folding upon heat stress. Notably, three of the four interaction-richest proteins (with more than 40 interactions) are cell-surface receptors (estrogen receptor 1, transforming growth factor beta receptor 1 and platelet derived growth factor receptor, beta) again suggesting a central role of signaling pathways for dynamic regulation in the liver (Figure 6D right).

### Protein Phosphatase 1 Modulates CLOCK/BMAL1-Dependent Transactivation

**Figure 7:**
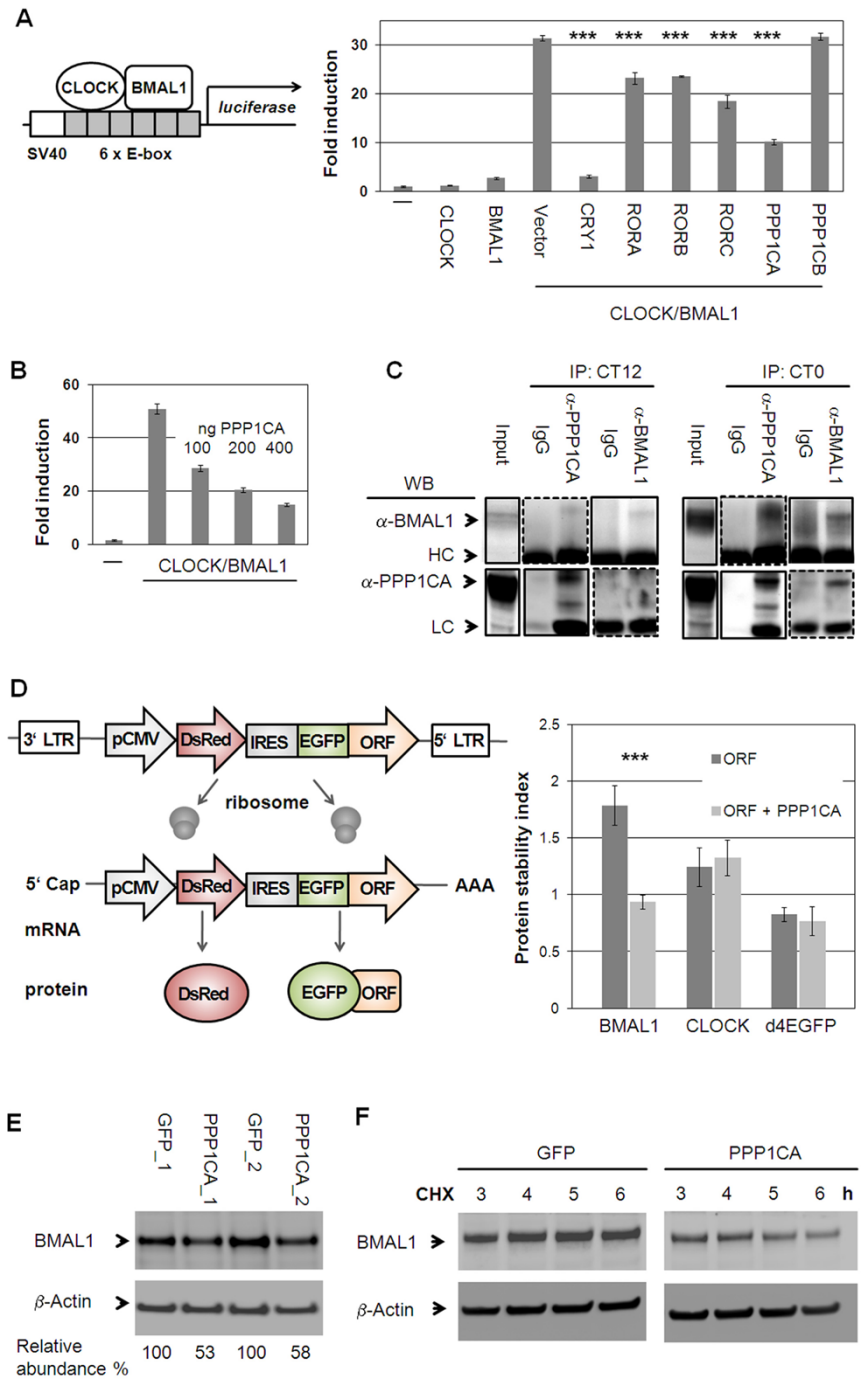
Protein Phosphatase 1 Modulates CLOCK/BMAL1 Function. (A) CLOCK and BMAL1 interactors identified in yeast and their paralogs were co-transfected with CLOCK/BMAL1 and an E-box containing luciferase reporter (see also Figure S7A). Shown are means ± s.d. of CLOCK/BMAL1 modifiers (n = 3; *** p<0.001, t-test). (B) PPP1CA dose-dependently reduces CLOCK/BMAL1 transactivation (n = 3; means ± SD.). (C) PPP1CA is present in the CLOCK/BMAL1 complex. Murine livers were harvested at indicated times. Dashed lines: longer exposure. (LC: light chain; HC: heavy chain). (D) PPP1CA destabilizes BMAL1 protein. Left: Stability is reported by the change of EGFP to DsRed ratio [30], [31]. Right: PPP1CA co-expression with BMAL1, CLOCK or short-lived EGFP fusion proteins in U2OS cells reduces BMAL1 stability (mean ± s.d.; ***p<0.001; t-test; n = 3; (see also Figure S7B, S7C). (E) Endogenous BMAL1 levels are reduced upon PPP1CA overexpression in U2OS cells. Depicted are two independent experiments. (F) PPP1CA reduces BMAL1 stability. U2OS cells stably expressing PPP1CA or GFP were harvested at the indicated time points after cycloheximide (CHX) application and protein levels were analyzed by Western blot. Shown is one representative of two independently performed experiments (see also Figure S7D).

Our systems biology analysis of the circadian interactome points to a timely regulated action of chromatin modifying enzymes (see Figure 6A, 6C). It is known that at the heart of the circadian oscillator binding of the transcription factor heterodimer CLOCK/BMAL1 is controlled by methylation and acetylation states of histones at specific promoter regions [21]–[25]. In addition, CLOCK/BMAL1 transactivation activity is modulated by a precisely timed acting repressor complex. In our Y2H screen, we discovered 15 new interaction partners for CLOCK/BMAL1, which might play a role in modulating their function in cells (see Table S1). We systematically tested whether these interactors (and/or their paralogs – in total 28) are able to modulate CLOCK/BMAL1 transactivation measured from an *E-box* containing artificial promoter with firefly luciferase as reporter (Figure S7A). As expected, already characterized CLOCK/BMAL1 repressors such as CRYs, PER2 and DECs [4], [26], [27] substantially inhibited transactivation. Interestingly, among the 15 new interactors (including their paralogs) PPP1Cα (protein phosphatase 1 alpha, catalytic subunit), but not PPP1Cβ severely and RORs moderately reduced the reporter signal (Figure 7A). The effect of PPP1Cα on CLOCK/BMAL1-mediated transactivation was dose dependent (Figure 7B).

Our *in silico* analysis predicted that PPP1Cα binds to the CLOCK/BMAL1 complex in mouse liver in a time-of-day specific manner. We tested this by co-immunoprecipitation experiments using antibodies against endogenous proteins (Figure 7C). We selected circadian times that were predicted by our dynamic interaction analysis (Figure 4B) to correspond to maximal and minimal likelihood of PPI. Indeed, we detected an association of endogenous PPP1Cα with CLOCK/BMAL1 at CT0 (CT = circadian time) while only little PPP1Cα-CLOCK/BMAL1 complex was found at CT12 suggesting a circadian time-dependent modulation on CLOCK/BMAL1 function. As CLOCK and BMAL1 phosphorylation have been described to affect their stability [28], [29], we tested whether PPP1Cα acts on this level. We stably expressed CLOCK and BMAL1 as a GFP-fusion protein in human U2OS cells with DsRed (a red fluorescent protein) on the same transcript [30], [31]. Protein stability can be monitored *via* the ratio of GFP to DsRed signal using FACS analysis thereby normalizing for different transcription rates in individual cells. As proof-of-concept of this approach, we confirmed the previously reported destabilizing effect of GSK3β on BMAL1 [32] (Figure S7B–S7D). While we could not detect an effect of PPP1Cα on CLOCK stability, we saw a substantial and significant decrease of BMAL1 abundance (Figure 7D). In addition, endogenous BMAL1 levels were reduced by about 50% upon stably overexpression of PPP1Cα in U2OS cells (Figure 7E). Lower BMAL1 abundance in the presence of PPP1Cα is likely due to reduced BMAL1 stability, since cycloheximide treated cells (in which *de novo* protein synthesis is blocked) revealed a much faster degradation of endogenous BMAL1 when PPP1Cα is overexpressed (Figure 7F). Together, these data indicate that BMAL1 stability and probably thereby transactivation is regulated by PPP1Cα.

## Discussion

### Novel Protein–Protein Interactions within the Molecular Oscillator

Protein–protein interactions among circadian clock proteins are often time-of-day dependent, which is crucial for the function of the molecular circadian oscillator. While the recent years have witnessed the identification of an increasing number of clock proteins or modulators [15], [16], [21], [33], [34] a comprehensive analysis of PPIs within the circadian clockwork - in particular with respect to the timing of the PPIs - is still missing. Here, we identified 109 so far uncharacterized interactions within the circadian clockwork in yeast and have successfully validated a sub-fraction in mammalian cells. While our matrix screen design allowed us to perform independent replica experiments thereby reducing the risk of false positives and false negatives, it is clear that due to the obvious limitations of the Y2H system [35] our network is likely still far away from saturation. For example, interactions that depend on posttranslational modifications or on more than two proteins are difficult to detect in Y2H assays. Nevertheless, our screen showed a rather high sensitivity (∼40% recovery of previously reported PPIs) compared to other Y2H reports or other PPI interaction methods [11]. In addition, we estimate to have only a low false-positive rate, since we could validate 86% of all CLOCK and BMAL1 interactions in mammalian cells.

Interestingly, many of the new interactions occurred between core clock components and regulatory components such as kinases *e.g*., CSNK2β, phosphatases (*e.g*., PPP2, PPP1), and F-box proteins (*e.g*. FBXW11). Hence, our data should be a valuable resource for studying molecular events within the circadian system with so far uncharacterized posttranslational mechanisms being especially interesting. Whereas phosphorylation of clock proteins are increasingly recognized as crucial for circadian dynamics, de-phosphorylation events have not been studied as extensively [36]. Therefore, we characterized the newly discovered interaction between PPP1Cα and the CLOCK/BMAL1 heterodimer. Indeed, we could validate our *in silico* prediction of the daytime-dependence of this PPI, which negatively regulates BMAL1 abundance (see Figure 7 and Figure S7), whereas others propose PER proteins as substrates of PPP1 [37], [38]. Further work is needed to identify the respective regulatory subunits that may mediate substrate specificity.

### Circadian Protein–Protein Networks

Our circadian PPI network is very densely connected (Figure 2) with a high clustering. How can such a network function? We analyzed both the predicted temporal organization, which separate PPIs in time as well as modular organization, which organize the network in functional complexes. To investigate temporal organization, we have integrated circadian expression profiles from mouse liver for the interacting pairs of proteins assuming that co-expression on transcript level can represent individual protein abundance probably as one limiting factor for physical interaction. De Lichtenberg et al. have pioneered the analysis of dynamic protein–protein interactions with a specific focus on cell-cycle stages in yeast also integrating transcription data [39] and Atwood et al. predicted the interaction time of circadian co- and antiphasic expressed proteins [40]. However, our analysis is not restricted to a specific process or specific circadian phases, but provides a systems-wide view of circadian PPI dynamics.

Our transcript-based analysis led to the construction of a dynamic circadian (albeit only liver-specific) PPI network, in which PPIs are formed at all circadian phases (see Figure 4B). Obviously, our analysis harbors several limitations, since PPIs *in vivo* depend on a variety of factors such as spatial restrains, restriction to specific tissues, relative protein abundance, mRNA processing, stoichiometry and interaction kinetics, complex formation and posttranslational modifications. All these parameters are not represented by the corresponding mRNA profiles of interaction partners. However, our assumption that indeed dynamic binding events can be approximated by such an approach is supported by (i) our finding that co-expression of transcripts at similar circadian phases more often occurs among interacting proteins (see Figure 4B and Figure S6B), (ii) known interaction dynamics between components of the circadian system can be reproduced (see Figure 4B), *e.g.* the circadian phase-specific CLOCK/CRY1 interaction [5], and (iii) the *in silico* predicted time-of-day dependent interaction between PPP1Cα and CLOCK/BMAL1 could be validated with endogenous liver components. Nevertheless, it should be noted, that on a systems-wide scale it is still largely unknown, whether and to which extent genes with rhythmically expressed transcripts also display circadian protein levels. While recent comparisons between transcript levels and protein levels have shown a rather good correlation [41], [42], our circadian PPI network should still be considered as a prediction.

Dynamic ‘hubs’ (proteins predicted in many rhythmic PPIs) seem to be especially important for circadian rhythms (see Figure 5C) as revealed by our genetic perturbation analyses. Thus, apparently not the absolute number of interactions is crucial for the importance of a clock protein but the degree of dynamic PPIs. This may be not too surprising, since precisely timed interactions between activators and their repressors is the fundamental principle of the circadian negative feedback mechanism. Interestingly, this principle may be translated to a global scale: we find that proteins with a rhythmic transcript have significantly more interaction partners than non-rhythmic proteins (p<10^−10^, Wilcoxon Rank test). In addition, proteins that qualify as regulatory components (as defined by their GO category ‘regulation of biological process’) have significantly more interaction partners than non-regulatory proteins (p<10^−15^, Wilcoxon Rank test; see also Text S1). Together, this suggests that rhythmic control of PPIs is an essential feature of biological networks. While such analyses are only of correlative nature, it would be interesting in future studies to analyze directly whether a particular PPI or the rhythmicity of a particular PPI is required for normal rhythms. To this end, however, novel (perhaps pharmacological) tools are needed to specifically disrupt the PPI without interfering with the abundance or other PPIs the component might execute.

### Regulation of Cellular Physiology by Dynamic Protein–Protein Interactions

In the last decade transcriptome analysis were successfully used to study circadian dynamics on a systems-wide level [6], [7] with mRNA rhythms serving as indicators for output control. Corresponding comprehensive studies on the level of the proteome are still largely missing. To get novel insights into the time-of-day dependent regulation of cellular processes we propose a new strategy to predict circadian regulation at the level of protein complexes rather than looking at mRNA profiles of individual components. Based on this dynamic interactome we have constructed a ‘process network’ with many processes (represented by corresponding GO terms) strongly connected by predicted dynamic PPIs (see Figure 6D and Figure S6C). While this concept has obvious limitations (ambiguous GO assignments, predictive nature of rhythmic PPIs, etc.) it allows a first, systems-wide glance on how cellular processes might be regulated in a time-of-day specific manner beyond circadian transcription. Future studies are needed to investigate to what extent and on what mechanistic bases rhythmic PPIs contribute to the dynamic modulation of cellular processes.

Overall, we propose a global view on the circadian control of protein–protein interactions important not only for the circadian oscillator but also for the temporal orchestration of many essential cellular processes.

## Materials and Methods

### Y2H Interaction Mapping

Matrix-based Y2H interaction analyses were performed essentially as described [10], [43]. For the generation of the Y2H matrix 46 full-length entry constructs were shuttled into Y2H vectors resulting in *LexA* DNA binding domain fusions (bait configuration) and *Gal4* transcription activation domain hybrids (prey configuration). The *L40ccαMATα* yeast strain was transformed with prey constructs while baits were introduced into a *MAT*a strain carrying *HIS3*, *URA3*, and *lacZ* as reporter genes. All constructs were tested for auto-activation properties. For mating, liquid cultures of the *MAT*a strain were mixed with prey colonies in 384-micro titer plates and mixtures were then spotted onto yeast complete medium agar plates. After mating at 30°C, colonies were transferred into 348 well plates containing SDII liquid (-Leu, -Trp) selective medium and then transferred to SDII agar for selection of diploid yeast (at 30°C). Diploid yeasts were spotted on solid selective SDIV agar plates (-Leu, -Trp, -Ura, -His) as well as on nylon membranes placed on SDIV agar plates. X-Gal assays were performed with the colonies that grew on membranes as described.

### Co-Immunopreciptitaion with BMAL1 or CLOCK-Luciferase Fusion Proteins

HEK293 cells were lentivirally transduced with *Clock-* or *Bmal1-luciferase* constructs. Cells stably expressing luciferase hybrids were transfected with MYC-tagged putative interactors. After 48 hours, lysate containing one million luciferase counts was subjected to immunoprecipitation. Pull-downs were performed with an anti-MYC or an isoform specific ideotypic antibody and agarose beads after overnight incubation. After three washes luciferase activity of pulled-down complexes was measured.

### Western Blot Analysis

Western blot analysis was performed essentially as described [15]. Briefly, proteins were denatured *via* boiling in SDS-loading buffer. Separation was performed by SDS-PAGE using 4%–12% Bis-Tris gels. Proteins were transferred to nitrocellulose membrane and incubated with primary antibodies. Membranes were probed with corresponding HRP-conjugated secondary antibodies. Chemiluminescence reaction was performed for protein visualization.

### Genetic Perturbation and Circadian Phenotyping

RNAi and overexpression constructs were lentivirally delivered as described [15]. Briefly, filtered medium containing virus particles was used for transduction of human U2OS cells carrying the *Bmal1*-promoter luciferase reporter [15] in the presence of protamine sulfate. Next day, medium was exchanged to puromycin or blasticidine containing medium. After positive selection cells were synchronized by a 30 min pulse of dexamethasone. Bioluminescence was monitored for ∼6 days in a TopCount luminometer with a sampling rate of 30 min. Time series were analyzed for circadian rhythmicity correlating them to a cosine function *via* the ChronoStar software [15].

### Co-Transactivation Assay

HEK293 cells were transiently transfected with a firefly luciferase reporter (containing six *E-box* enhancer elements), CLOCK/BMAL1 and individually all discovered putative CLOCK and BMAL1 interactors (including their paralogs or functional subunits) and a renilla luciferase construct for normalization [4], [27]. Signals were detected with a dual-luciferase reporter assay in a luminometer plate reader. Experiments were repeated three times.

### Protein Stability Measurement of EGFP-BMAL1

U2OS cells stably expressing a fluorescence reporter either with BMAL1 or CLOCK as EGFP fusion protein (see Figure 7D left; [30], [31]) were transduced with lentiviruses containing PPP1Cα or GSK3β expression constructs. Cell fluorescence was analyzed using flow cytometry (FACS Canto II). Red fluorescence of DsRed and green fluorescence of EGFP intensities of DsRed positive cells were detected. The protein stability index (PSI) is defined as the maximum of the distribution curve of the ratio between EGFP and DsRed intensities. Thus, a high PSI value corresponds to a high green fluorescence intensity, *i.e.* highly abundant (and likely stable) fusion protein.

### Prediction of Rhythmic Protein–Protein Interactions

Firstly, standardized 48-hour transcript liver profiles taken from Hughes et al., 2009 [6] were analyzed for 24 hour periodicity using Fourier analysis:
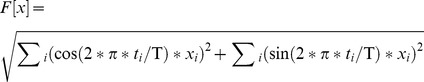
where ***x*** is the standardized expression vector (mean(***x***) = 0; sd(***x***) = 1) for the gene, T is the period (in our case 24 h), and *x_i_* is the measured expression at time point *t_i_*. Statistical significance was calculated by comparison with randomly permutated time series using the Bioconductor cycle package [44]. Secondly, abundance *A_C_* of a complex *C* formed by two interacting proteins *P*_1,2_ is assumed to be proportional to the expression *E* of *P*_1,2_. Abundance *A_C_(t)* over time is approximated by the product of expression vectors *E*_P1_(t) * *E*_P2_(t), which was then associated to the corresponding PPI. As proxy for protein abundance, the transcript levels over time were utilized, thus
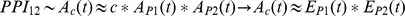
Statistical significance of *A_C_(t)* rhythmicity was calculated using the Fourier-score and permutated time series as background model after standardization (*i.e.* mean (*E_P_*_1_ * *E_P_*_2_) = 0; sd (*E_P_*_1_ * *E_P_*_2_) = 1). A phase was assigned to a periodic interaction through shifting a cosine (with 24 h periodicity) along the time axis and measuring the overlap of the expression levels with the cosine curve. The time shift leading to a maximum overlap was considered as the phase α of the PPI and ranges from 0 to 24 h.

### Construction of the Dynamic Interactome

All PPIs of the compiled human interactome in the UniHI database (N = 45775) were assessed for possible dynamic behavior [12], [45], as described above. Human proteins were mapped to their mouse orthologs and periodicity of 30413 interactions was analyzed as described above resulting in the prediction of 2788 significantly (FDR<10^−5^) dynamic interactions.

### Data Availability

The discovered PPIs are listed in the IMEx (http://www.imexconsortium.org) consortium through IntAct [pmid: 19850723] and assigned the identifier IM-16832.

## Supporting Information

Figure S1 The Yeast-2-Hybrid (Y2H) Approach (referring to Figure 1A–1C). (A) Principle of Y2H screen. Y2H is a genetic approach where two interacting proteins can reconstruct a functional transcription factor, which leads to the activation of several reporter genes (*ura*, *his*: growth selection on minimal media; *lacZ*: β-Galactosidase activity). One interactor X is fused to a DNA binding domain (DBD: LEXA; bait configuration), while the other Y to a transcription activation domain (AD: GAL4; prey configuration). Both hybrids are transformed into different yeast strains (MATa or MATα). Interaction of X and Y is detected after mating *via* activation of reporter genes. (B) Left: matrix-based Y2H screening. Defined bait and prey fusions allow performing several repetitions of an interaction screen. Matrix position reveals positive interaction pairs without the necessity of sequence identification. Right: library-based Y2H screening. Bait is presented to prey library, which may contain redundant sequences. Growth competition and sequencing are required for the identification of interactors (modified from Golemis E.A. and Adams P.D. (2005) Protein–protein Interactions 2^nd^ ed. New York: CSHL PRESS. 744p). (C) Auto-activation test for 46 circadian clock components. Left: yeast strain containing preys were mated with a yeast strain expressing the DBD-domain only. Results are shown for four independent mating experiments. NPAS2 (position D2) showed weak auto-activating properties (weak growth on selective media, no *lacZ* expression) but was nonetheless included for high-throughput interaction mapping. Right: auto-activation test for baits. Yeast containing baits were mated with a yeast strain expressing the AD-domain only. PER2 (A2), BMAL1 (A5), NR1D1 (B5), NR1D2 (B6), RORB (C3) and PPP2CA (G1) showed strong auto-activation of all reporters leading to exclusion of these components in the bait configuration from further interaction experiments.
(TIF)

Figure S2 Reproduction Rate of Y2H Screen and Validation of CLOCK and BMAL1-Interactions in Mammalian Cells (referring to Figure 1C, 1D). (A) 109 interactions that occurred in our matrix-based Y2H screen were so far uncharacterized. 23 interactions were previously found in library based Y2H screens and reproduced with our approach, whereas 18 interactions were reproduced that were detected previously by other approaches (not in yeast cells) (for reproduced interactions see Table S1). 63 previously detected PPIs were not found in this Y2H screen. (B) Principle of co-immunoprecipitation experiments. HEK293 cells stably expressing CLOCK or BMAL1 C-terminal luciferase fusions were transfected with MYC-tagged interactors. Lysates containing one million luciferase counts were subjected to immunoprecipitation experiments. Pull-downs were preformed with an anti-MYC antibody or an ideotypic antibody in (beads) controls. After washing, beads pellets were incubated with a luciferin containing reagent and luciferase activity was measured (for details see Text S1). (C) Input detection via Western blot analysis. 25 µg of total lysate were loaded per lane as an input control. MYC-fusions were detected with an anti-MYC antibody. The results for the co-immunoprercipitations as performed in Figure 1D are shown. CLOCK and DBP fusions could not be detected in lysates as MYC (*), FLAG or V5-hybrids (not shown). Expected protein size (from SwissProt database (www.expasy.org)) is shown in brackets. (D) NONO and NR1D2 were not detected as direct BMAL1 interactors in yeast. Co-immunopreciptitation experiments as performed for validation in Figure 1D with BMAL1-LUC and MYC-NONO or MYC-NR1D2 also show no interaction in mammalian cells using our validation system. Western blots show input controls as performed in (C).
(TIF)

Figure S3 Construction of the Circadian Protein–protein Interaction Network and Topology Analysis (referring to Figure 2). (A) Enrichment and extension of the circadian protein–protein interaction network. The experimental derived network was firstly enriched by adding 63 previously described interactions (Y2H screen false negatives) from literature and extended by 88 direct neighbors of clock core components as stored in the UniHI database. This resulted in the construction of a circadian protein–protein interaction network consisting of 134 proteins and 625 interactions (see also Table S1). (B) Degree frequency of proteins in the circadian clock network. The number of proteins was plotted as a function of the number of neighbors that proteins in the network have. The degree frequency indicates properties of a ‘scale-free’ network, *i.e.* many proteins have few and few proteins have many interactions. (C) Dependence of the clustering coefficient [10] on the number of interactions of proteins. The clustering coefficients were derived by averaging over all proteins with the same number of interactions (degree). The linear fit of the logged values is shown as solid line. (D) Dependence of the topological coefficient [10] on the number of interactions of proteins. The displayed topological coefficients were derived by averaging over all proteins with the same degree. The linear fit of the logged values is represented as solid line.
(TIF)

Figure S4 Visualization of Altered Circadian Phenotypes for Clock Core and Regulatory Components Upon Genetic Perturbation (referring to Figure 5A, 5B). (A) Systematic gene silencing. RNAi constructs were lentivirally delivered into U2OS cells carrying the *Bmal1*-promoter luciferase reporter and oscillation dynamics were monitored for several days. Data were detrended using the Chronostar analysis software. Black lines show non-silencing controls. Red dotted lines depict phenotypes for one RNAi construct. Period differences from mean are given (ar: arrhythmic, amp: low amplitude, damp: high damping, mag: magnitude). (B) Systematic overexpression. GFP overexpression was used as controls (black curves) Phenotypes were visualized as described in (A).
(TIF)

Figure S5 Characterization of Hubs in the Circadian Protein–protein Interaction Network (referring to Figure 4). Background distribution of average Pearson correlation coefficient (PCC) for fully random drawn interactions partners (black line) and partially random interaction partners (blue line) as well as the observed average PCC for 4 proteins in the circadian network are shown. CRY2 obtained significantly higher average PCC (FDR<0.01) than expected by chance whereas AR and CLOCK obtained significantly lower average PCC (FDR<0.01) than expected by chance. The PCC of ARNTL/BMAL1 is not significantly altered.
(TIF)

Figure S6 Identification of Functional Modules within the Circadian Protein–protein Interaction Network and Dynamic Regulation within the Global Interactome (referring to Figure 6C, 6D). (A) Highly connected clusters were identified using the Cytoscape plugins MCODE or the ClusterOne algorithm (for details on analysis see Text S1). Node colors: grey – network neighborhood, red – clock core, green – regulatory components. Yellow circles highlight rhythmic RNA profiles. Numbers are mRNA peak times in circadian time (CT). Modules were analyzed for enrichment of processes using Gene ontology (GO), KEGG and Pfam family annotations (see also main text as well as Figure 6C and Tex S1 for significance of enrichment). (B) Construction of a global dynamic protein–protein interactome. (C) Coupling of biological processes within the interactome *via* predicted rhythmic PPIs. Significance of connections was calculated based on the comparison with randomized versions of the dynamic interactome. Connections, for which no more than 10 out of 1000 random networks show a larger number of predicted rhythmic interactions are displayed. In total, 26 processes are linked *via* 52 connections. Node color: significance of enrichment in components with dynamic interactions (yellow: p<0.25; orange: p<10^−4^; red: p<10^−8^); node size: number of genes per category; edge color: number of random networks with larger number of rhythmic interactions between processes (blue: N = 0; green: N≤10); edge width: number of rhythmic interactions. Processes, for which N≤10 random networks have more internal dynamic interactions than observed in the global interaction network, were highlighted with a dark red border.
(TIF)

Figure S7 Protein Phosphatase 1 Modifies BMAL1 Abundance (referring to Figure 7). (A) Systematic screen for new modulators of CLOCK/BMAL1 transactivation. All CLOCK and BMAL1 interactors identified in our Y2H experiments and their paralogs were co-transfected with CLOCK/BMAL1 together with an artificial 6 *E-box-Luciferase* containing reporter. Normalization was performed to Renilla-Luciferase signal. Shown is one representative result (n = 3; ± s.d.) of three independent experiments. Among new CLOCK/BMAL1 interactors RORs and PPP1CA showed consistent suppression of CLOCK/BMAL1 transactivation (see also Figure 7A, 7B), while increase of transactivation upon coexpression of casein kinase 1α/δ and WDR5 was not detected in all three experiments (B) GSK3B affects BMAL1 stability. Effect of GSK3B overexpression in U2OS cells also expressing either BMAL1 or short-lived EGFP control (d4EGFP) fusion proteins in the reporter construct. Left panel: protein stability index representing the peak of the distribution of the ratio between EGFP and DsRed fluorescence intensities (representative result of three independent measurements; average ± s.d.; n = 3 per condition; ** p<0.001); Middle panel: Distribution plots of the ratio EGFP to DsRed fluorescence (average ± s.d.; n = 3 per condition). Right panel: Representative green fluorescence (y-axis) *vs.* red fluorescence (x-axis) dotplots of flow cytometry analysis. Red gates encircle cell distributions of indicated ORF without addition of GSK3B. (C) Similar experiment as described in (B) with U2OS cells overexpressing PPP1CA or control (see also Figure 7B). (D) PPP1CA overexpression efficiency in lentivirally transduced U2OS cells (see also Figure 7E and 7F).
(TIF)

Table S1 List of PPIs among circadian clock proteins as well as enrichment and extension of the circadian PPI network (refers to Figure 1 and Figure 2).
(XLSX)

Table S2 Circadian phenotypes of oscillating U2OS cells upon RNAi-mediated downregulation of genes in the PPI network neighborhood (refers to Figure 3).
(XLSX)

Table S3 List of rhythmic PPIs within the circadian network as well as ‘hub’ analysis (refers to Figure 4B and Figure S5).
(XLSX)

Table S4 Dynamic degree and phenotypic score of circadian components (refers to Figure 5).
(XLSX)

Table S5 Coupling of biological processes via predicted dynamic PPIs (refers to Figure 6).
(XLSX)

Text S1 Supporting materials and methods.
(DOCX)
