# Supplementary figures and images for: Dynamic Circadian Protein–Protein Interaction Networks Predict Temporal Organization of Cellular Functions
Source: PLoS Genet. 2013 Mar 28;9(3):e1003398. doi: 10.1371/journal.pgen.1003398 (PMC3610820; doi:10.1371/journal.pgen.1003398)

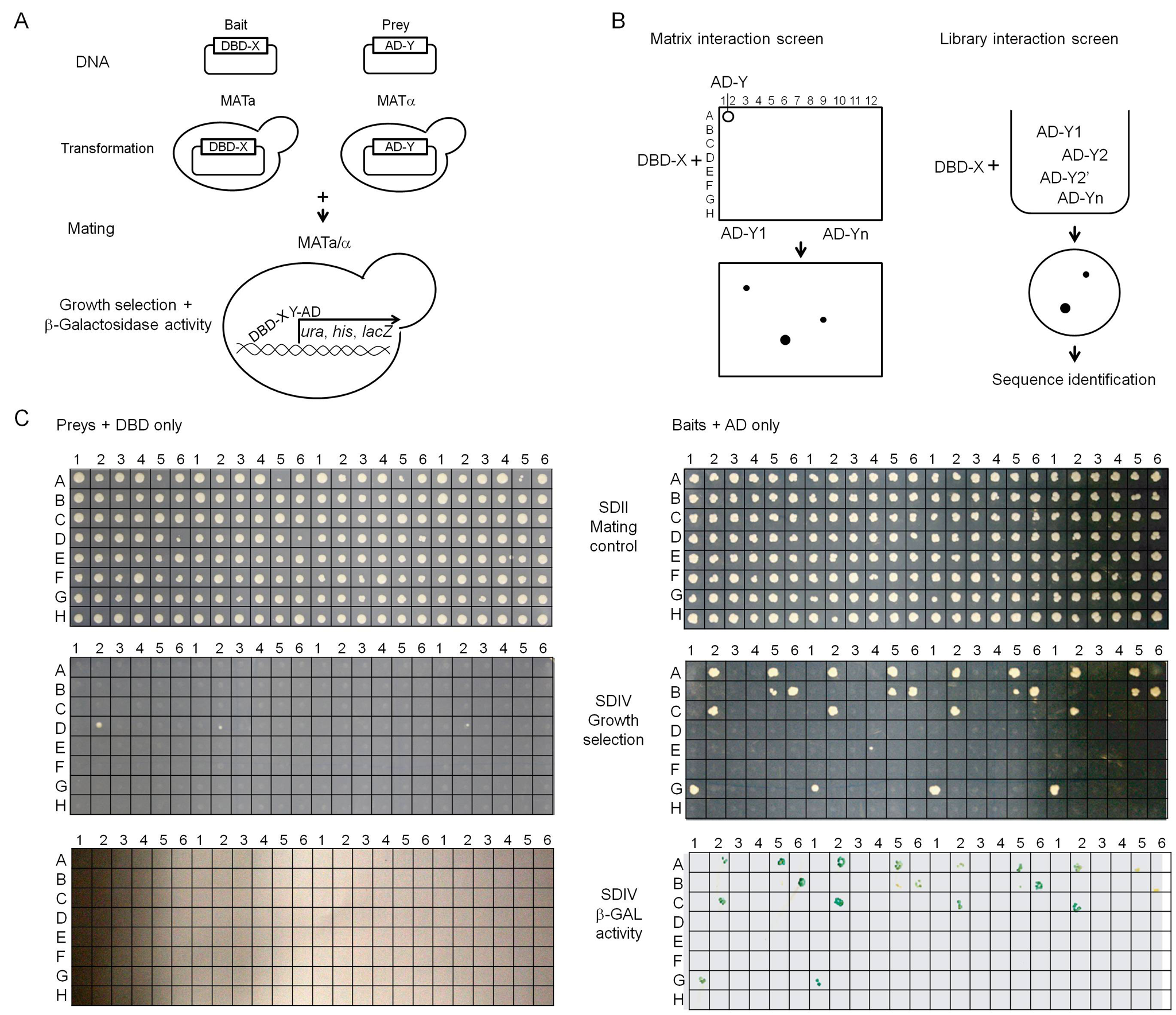

Supplement: Figure S1 — The Yeast-2-Hybrid (Y2H) Approach (referring to Figure 1A–1C). (A) Principle of Y2H screen. Y2H is a genetic approach where two interacting proteins can reconstruct a functional transcription factor, which leads to the activation of several reporter genes (ura, his: growth selection on minimal media; lacZ: β-Galactosidase activity). One interactor X is fused to a DNA binding domain (DBD: LEXA; bait configuration), while the other Y to a transcription activation domain (AD: GAL4; prey configuration). Both hybrids are transformed into different yeast strains (MATa or MATα). Interaction of X and Y is detected after mating via activation of reporter genes. (B) Left: matrix-based Y2H screening. Defined bait and prey fusions allow performing several repetitions of an interaction screen. Matrix position reveals positive interaction pairs without the necessity of sequence identification. Right: library-based Y2H screening. Bait is presented to prey library, which may contain redundant sequences. Growth competition and sequencing are required for the identification of interactors (modified from Golemis E.A. and Adams P.D. (2005) Protein–protein Interactions 2nd ed. New York: CSHL PRESS. 744p). (C) Auto-activation test for 46 circadian clock components. Left: yeast strain containing preys were mated with a yeast strain expressing the DBD-domain only. Results are shown for four independent mating experiments. NPAS2 (position D2) showed weak auto-activating properties (weak growth on selective media, no lacZ expression) but was nonetheless included for high-throughput interaction mapping. Right: auto-activation test for baits. Yeast containing baits were mated with a yeast strain expressing the AD-domain only. PER2 (A2), BMAL1 (A5), NR1D1 (B5), NR1D2 (B6), RORB (C3) and PPP2CA (G1) showed strong auto-activation of all reporters leading to exclusion of these components in the bait configuration from further interaction experiments. (TIF) [file pgen.1003398.s001.tif]

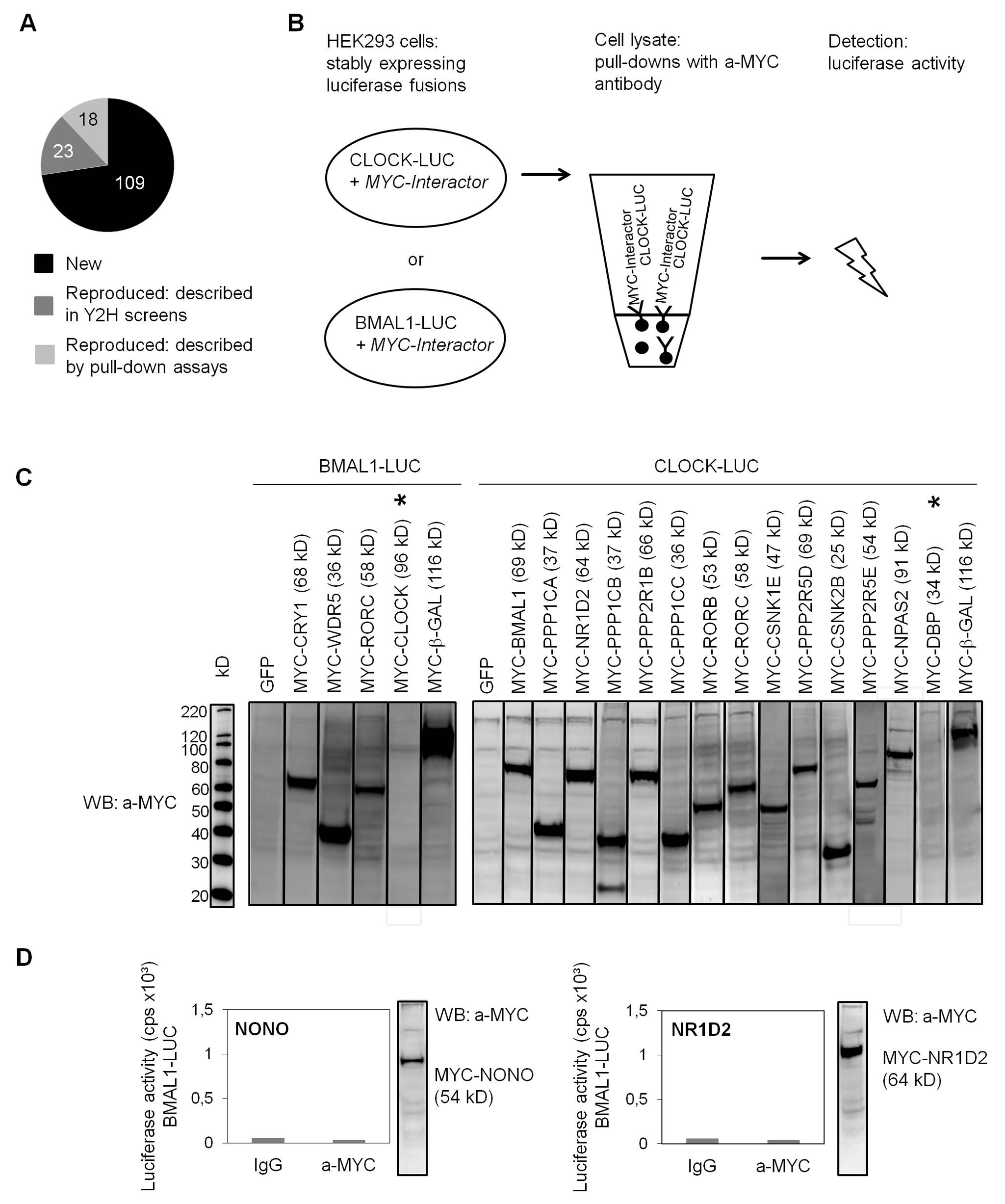

Supplement: Figure S2 — Reproduction Rate of Y2H Screen and Validation of CLOCK and BMAL1-Interactions in Mammalian Cells (referring to Figure 1C, 1D). (A) 109 interactions that occurred in our matrix-based Y2H screen were so far uncharacterized. 23 interactions were previously found in library based Y2H screens and reproduced with our approach, whereas 18 interactions were reproduced that were detected previously by other approaches (not in yeast cells) (for reproduced interactions see Table S1). 63 previously detected PPIs were not found in this Y2H screen. (B) Principle of co-immunoprecipitation experiments. HEK293 cells stably expressing CLOCK or BMAL1 C-terminal luciferase fusions were transfected with MYC-tagged interactors. Lysates containing one million luciferase counts were subjected to immunoprecipitation experiments. Pull-downs were preformed with an anti-MYC antibody or an ideotypic antibody in (beads) controls. After washing, beads pellets were incubated with a luciferin containing reagent and luciferase activity was measured (for details see Text S1). (C) Input detection via Western blot analysis. 25 µg of total lysate were loaded per lane as an input control. MYC-fusions were detected with an anti-MYC antibody. The results for the co-immunoprercipitations as performed in Figure 1D are shown. CLOCK and DBP fusions could not be detected in lysates as MYC (*), FLAG or V5-hybrids (not shown). Expected protein size (from SwissProt database (www.expasy.org)) is shown in brackets. (D) NONO and NR1D2 were not detected as direct BMAL1 interactors in yeast. Co-immunopreciptitation experiments as performed for validation in Figure 1D with BMAL1-LUC and MYC-NONO or MYC-NR1D2 also show no interaction in mammalian cells using our validation system. Western blots show input controls as performed in (C). (TIF) [file pgen.1003398.s002.tif]

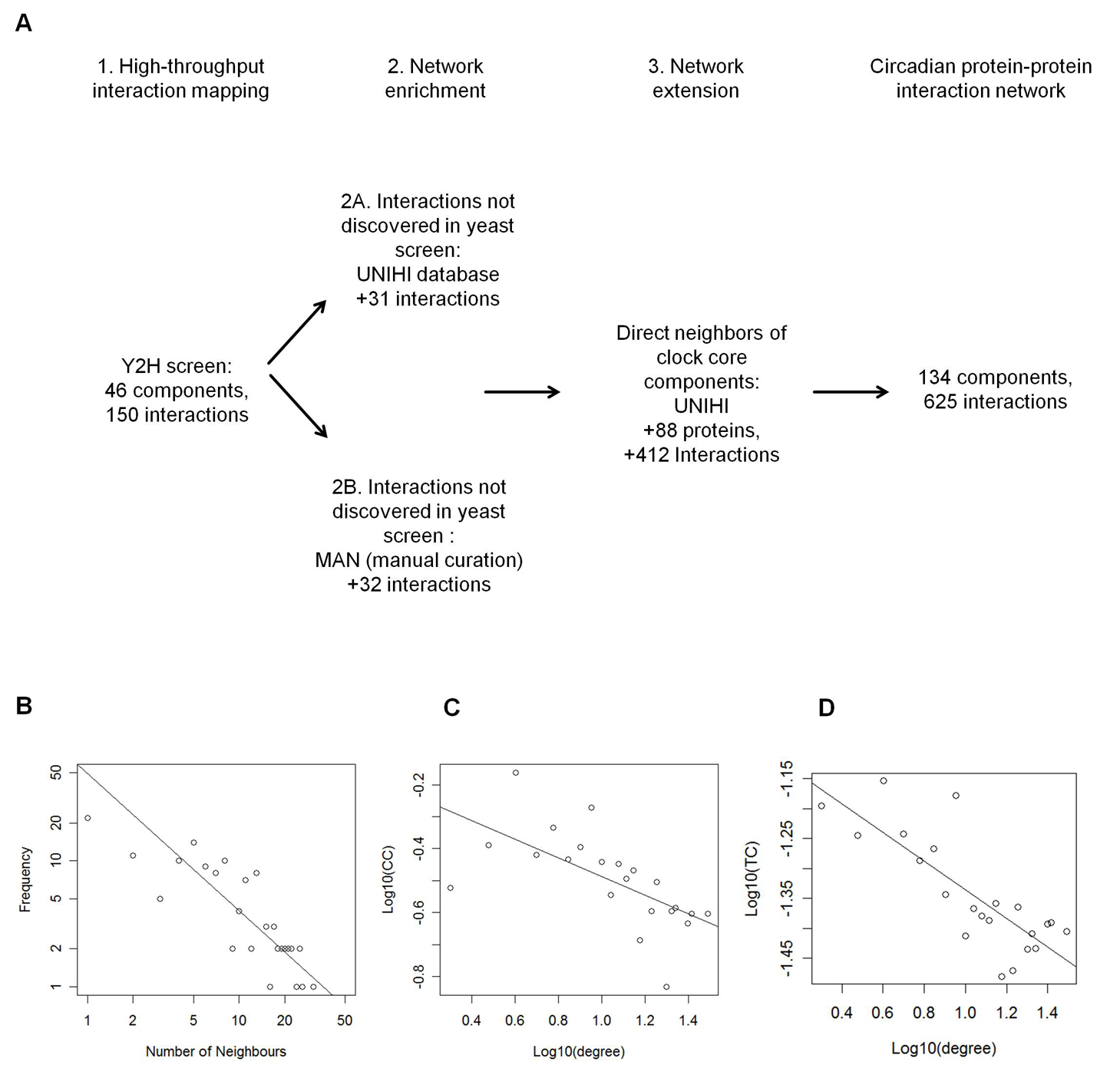

Supplement: Figure S3 — Construction of the Circadian Protein–protein Interaction Network and Topology Analysis (referring to Figure 2). (A) Enrichment and extension of the circadian protein–protein interaction network. The experimental derived network was firstly enriched by adding 63 previously described interactions (Y2H screen false negatives) from literature and extended by 88 direct neighbors of clock core components as stored in the UniHI database. This resulted in the construction of a circadian protein–protein interaction network consisting of 134 proteins and 625 interactions (see also Table S1). (B) Degree frequency of proteins in the circadian clock network. The number of proteins was plotted as a function of the number of neighbors that proteins in the network have. The degree frequency indicates properties of a ‘scale-free’ network, i.e. many proteins have few and few proteins have many interactions. (C) Dependence of the clustering coefficient [10] on the number of interactions of proteins. The clustering coefficients were derived by averaging over all proteins with the same number of interactions (degree). The linear fit of the logged values is shown as solid line. (D) Dependence of the topological coefficient [10] on the number of interactions of proteins. The displayed topological coefficients were derived by averaging over all proteins with the same degree. The linear fit of the logged values is represented as solid line. (TIF) [file pgen.1003398.s003.tif]

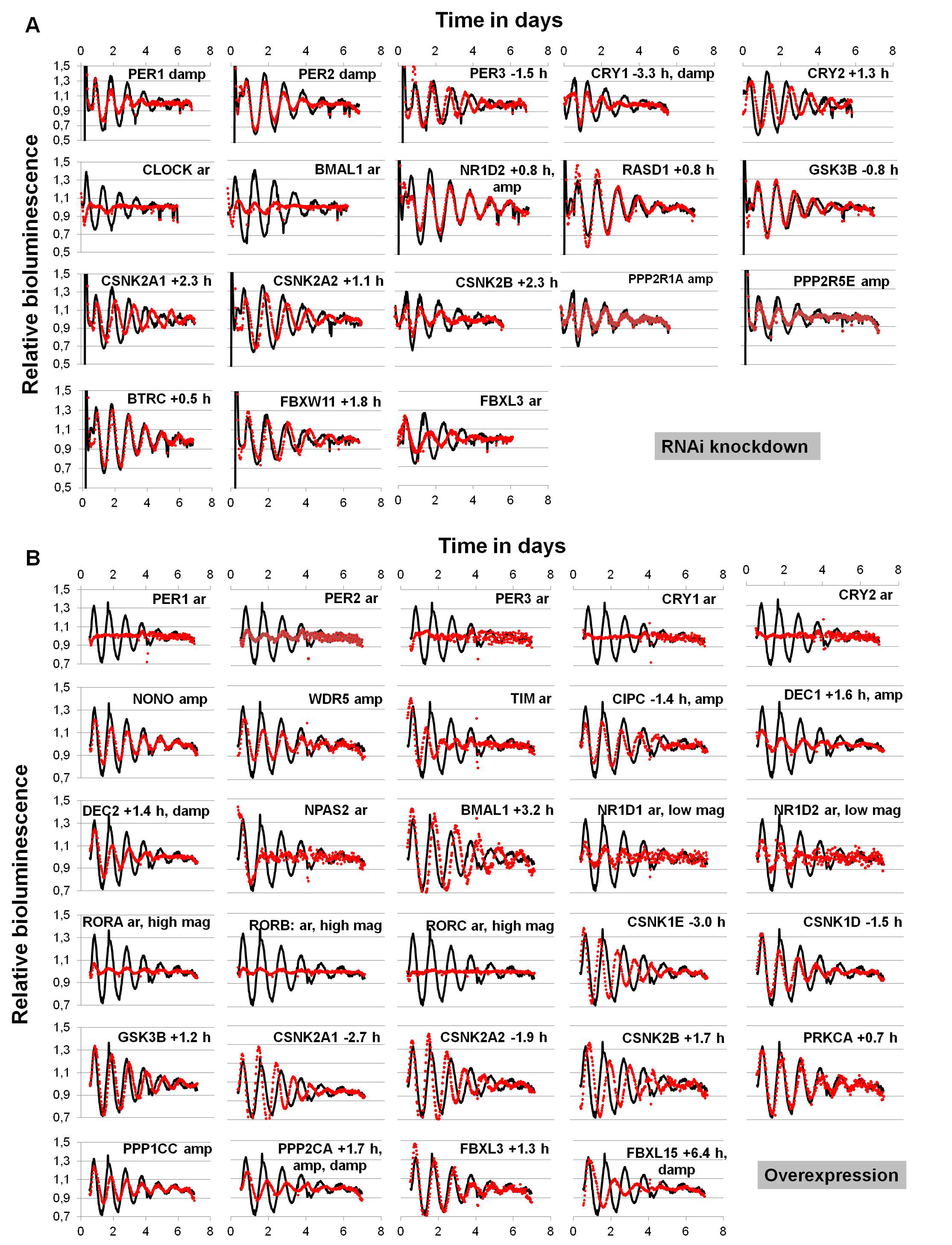

Supplement: Figure S4 — Visualization of Altered Circadian Phenotypes for Clock Core and Regulatory Components Upon Genetic Perturbation (referring to Figure 5A, 5B). (A) Systematic gene silencing. RNAi constructs were lentivirally delivered into U2OS cells carrying the Bmal1-promoter luciferase reporter and oscillation dynamics were monitored for several days. Data were detrended using the Chronostar analysis software. Black lines show non-silencing controls. Red dotted lines depict phenotypes for one RNAi construct. Period differences from mean are given (ar: arrhythmic, amp: low amplitude, damp: high damping, mag: magnitude). (B) Systematic overexpression. GFP overexpression was used as controls (black curves) Phenotypes were visualized as described in (A). (TIF) [file pgen.1003398.s004.tif]

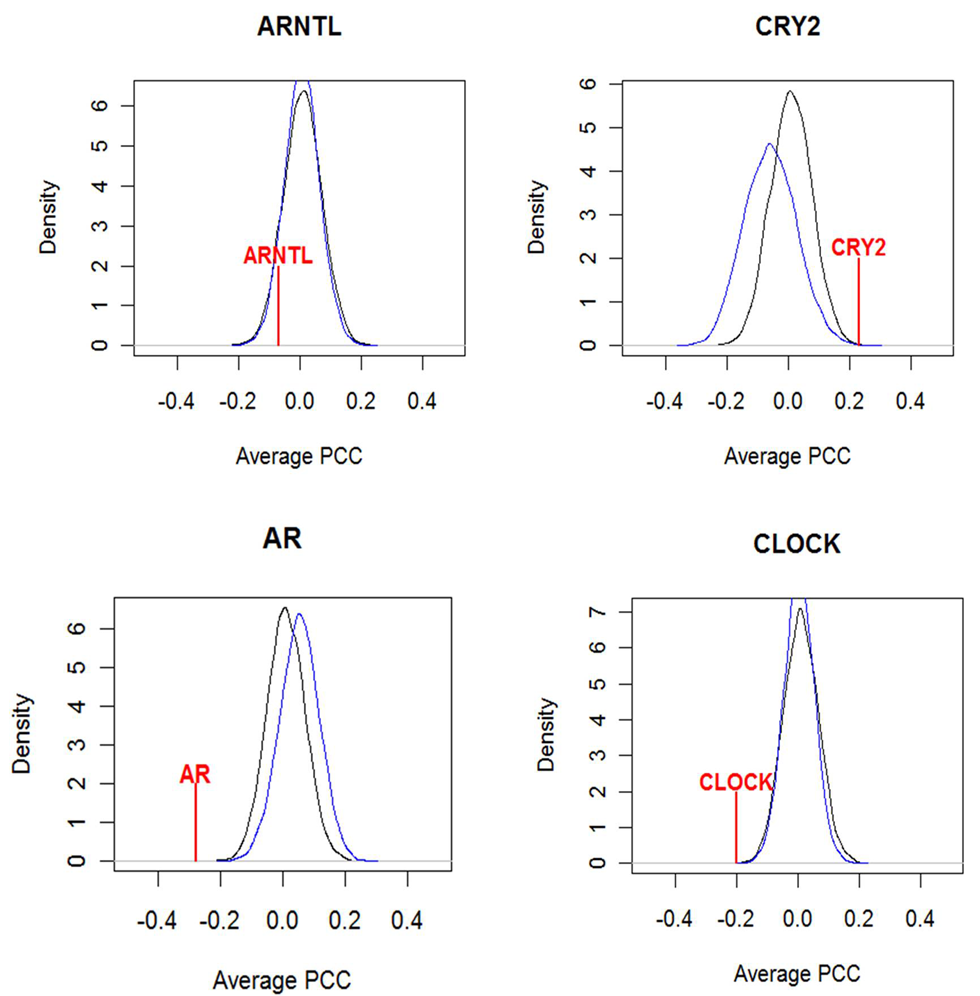

Supplement: Figure S5 — Characterization of Hubs in the Circadian Protein–protein Interaction Network (referring to Figure 4). Background distribution of average Pearson correlation coefficient (PCC) for fully random drawn interactions partners (black line) and partially random interaction partners (blue line) as well as the observed average PCC for 4 proteins in the circadian network are shown. CRY2 obtained significantly higher average PCC (FDR<0.01) than expected by chance whereas AR and CLOCK obtained significantly lower average PCC (FDR<0.01) than expected by chance. The PCC of ARNTL/BMAL1 is not significantly altered. (TIF) [file pgen.1003398.s005.tif]

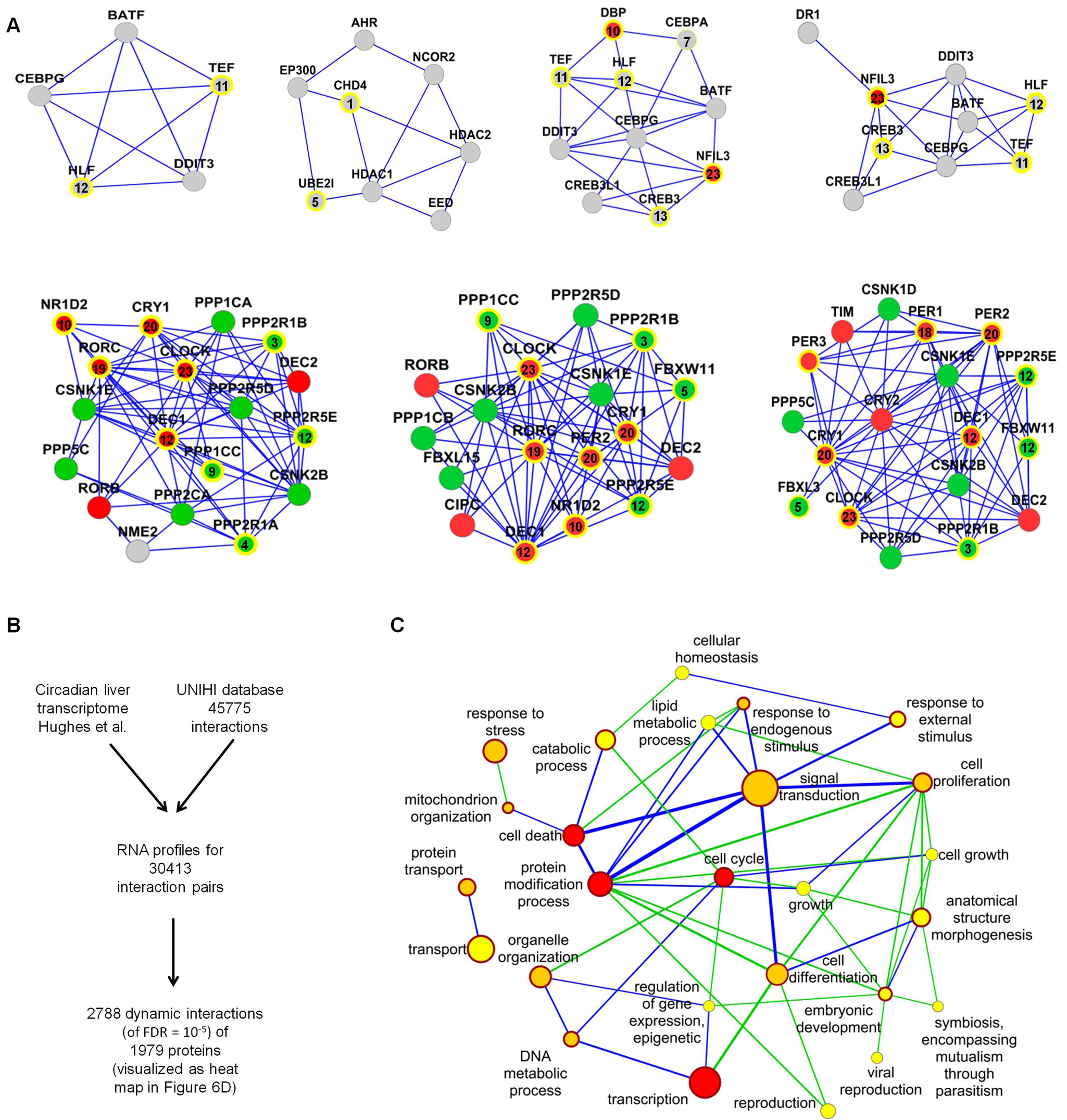

Supplement: Figure S6 — Identification of Functional Modules within the Circadian Protein–protein Interaction Network and Dynamic Regulation within the Global Interactome (referring to Figure 6C, 6D). (A) Highly connected clusters were identified using the Cytoscape plugins MCODE or the ClusterOne algorithm (for details on analysis see Text S1). Node colors: grey – network neighborhood, red – clock core, green – regulatory components. Yellow circles highlight rhythmic RNA profiles. Numbers are mRNA peak times in circadian time (CT). Modules were analyzed for enrichment of processes using Gene ontology (GO), KEGG and Pfam family annotations (see also main text as well as Figure 6C and Tex S1 for significance of enrichment). (B) Construction of a global dynamic protein–protein interactome. (C) Coupling of biological processes within the interactome via predicted rhythmic PPIs. Significance of connections was calculated based on the comparison with randomized versions of the dynamic interactome. Connections, for which no more than 10 out of 1000 random networks show a larger number of predicted rhythmic interactions are displayed. In total, 26 processes are linked via 52 connections. Node color: significance of enrichment in components with dynamic interactions (yellow: p<0.25; orange: p<10−4; red: p<10−8); node size: number of genes per category; edge color: number of random networks with larger number of rhythmic interactions between processes (blue: N = 0; green: N≤10); edge width: number of rhythmic interactions. Processes, for which N≤10 random networks have more internal dynamic interactions than observed in the global interaction network, were highlighted with a dark red border. (TIF) [file pgen.1003398.s006.tif]

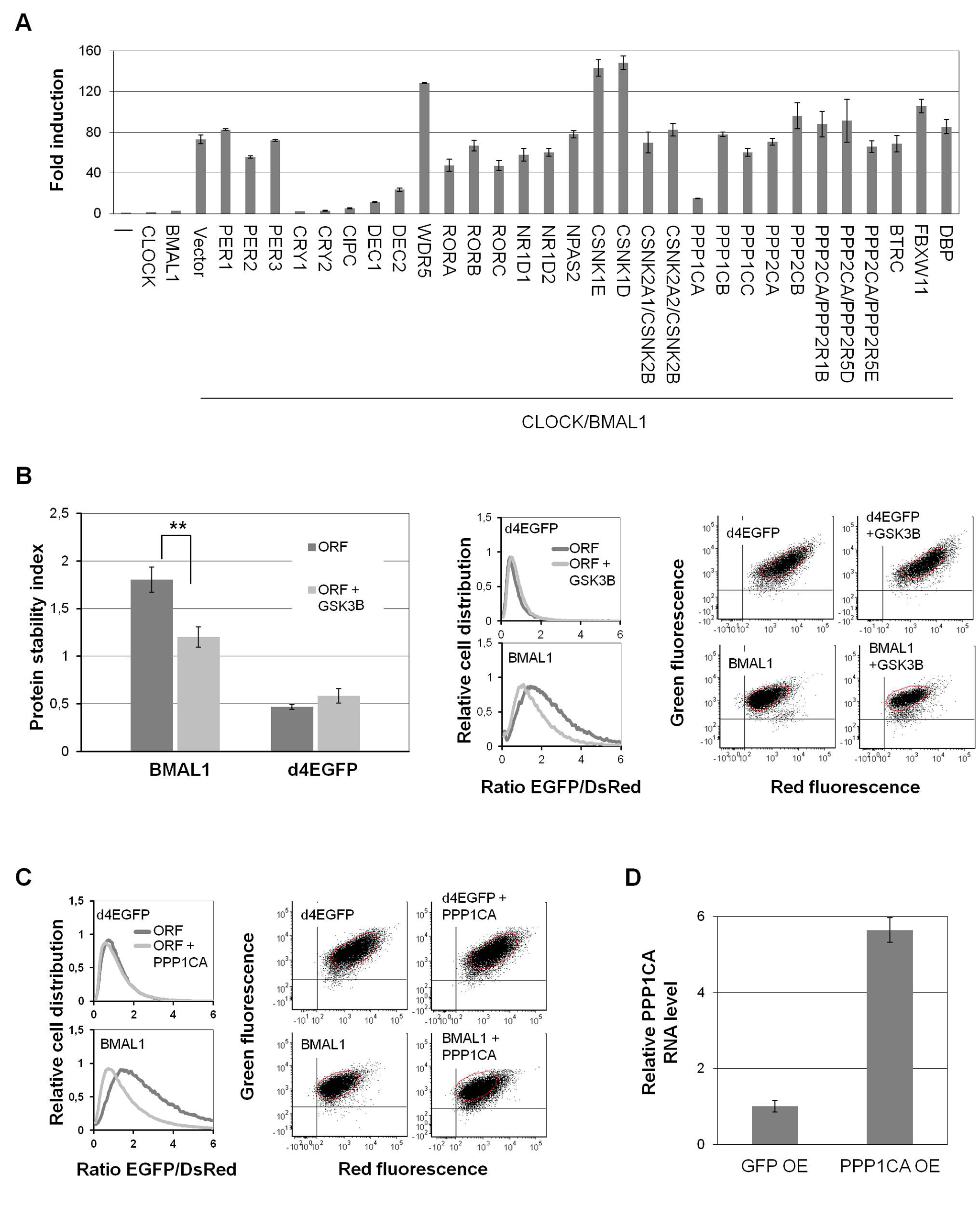

Supplement: Figure S7 — Protein Phosphatase 1 Modifies BMAL1 Abundance (referring to Figure 7). (A) Systematic screen for new modulators of CLOCK/BMAL1 transactivation. All CLOCK and BMAL1 interactors identified in our Y2H experiments and their paralogs were co-transfected with CLOCK/BMAL1 together with an artificial 6 E-box-Luciferase containing reporter. Normalization was performed to Renilla-Luciferase signal. Shown is one representative result (n = 3; ± s.d.) of three independent experiments. Among new CLOCK/BMAL1 interactors RORs and PPP1CA showed consistent suppression of CLOCK/BMAL1 transactivation (see also Figure 7A, 7B), while increase of transactivation upon coexpression of casein kinase 1α/δ and WDR5 was not detected in all three experiments (B) GSK3B affects BMAL1 stability. Effect of GSK3B overexpression in U2OS cells also expressing either BMAL1 or short-lived EGFP control (d4EGFP) fusion proteins in the reporter construct. Left panel: protein stability index representing the peak of the distribution of the ratio between EGFP and DsRed fluorescence intensities (representative result of three independent measurements; average ± s.d.; n = 3 per condition; ** p<0.001); Middle panel: Distribution plots of the ratio EGFP to DsRed fluorescence (average ± s.d.; n = 3 per condition). Right panel: Representative green fluorescence (y-axis) vs. red fluorescence (x-axis) dotplots of flow cytometry analysis. Red gates encircle cell distributions of indicated ORF without addition of GSK3B. (C) Similar experiment as described in (B) with U2OS cells overexpressing PPP1CA or control (see also Figure 7B). (D) PPP1CA overexpression efficiency in lentivirally transduced U2OS cells (see also Figure 7E and 7F). (TIF) [file pgen.1003398.s007.tif]
