# Supplementary material for: Dynamic Circadian Protein–Protein Interaction Networks Predict Temporal Organization of Cellular Functions
Source: PLoS Genet. 2013 Mar 28;9(3):e1003398. doi: 10.1371/journal.pgen.1003398 (PMC3610820; doi:10.1371/journal.pgen.1003398)
Supplement: Text S1 — Supporting materials and methods. (DOCX) [file pgen.1003398.s013.docx]

**Text S1 Supporting materials and methods**

**I. Experimental Procedures**

**1. High-Throughput Interaction Mapping**

**1.1 Selection of Candidates for the Yeast-Two-Hybrid (Y2H) High-Throughput Interaction Mapping (referring to Figure 1A**-**C)**

For the generation of the circadian protein-protein interaction network, all currently described circadian as well as assumed components in mammals were considered at the start of the study (July 2007). Furthermore, suggested candidates based on gene homology and orthology from other species (*Drosophila*, *Neurospora*) were included. *E.g*. to complete the set of well- established kinases within the molecular oscillator like CSNK1ε/δ and GSK3β [1], recent studies indicated the involvement of the mammalian PRKCα [2] and PRKACα described in the *Neurospora* system [3] to be important to regulate circadian rhythms. In addition, based on at this time unpublished findings of [4] CSNK2 subunits as well as FBXL3 [4-7] were also considered for systematic interaction mapping. *Fbxl15*, the mammalian homolog of *Drosophila* *Jetlag* [8] and the long isoform of mammalian TIMELESS (TIM) [9] were also selected as potential candidates. PPP2 catalytic subunits and the mammalian regulatory subunits closely related to *Drosophila Widerborst* were as well included for high-throughput interaction experiments (for list of candidates see below).

**1.2 Generation of the Y2H Matrix (referring to Figure 1A)**

17 circadian clock genes were amplified *via* PCR and TOPO cloning (Invitrogen) was performed according to the manufacturer’s protocol to generate a Gateway^TM^ compatible entry clone collection. Primer pairs and source of additional 29 entry constructs are shown below. All 46 open reading frames (ORFs) were sub-cloned into bait (pBTM116-D9) and the prey destination vectors (pACT4-DM), respectively [10,11]. DNA quality was controlled by digestion in the attR1/attR2 sites of yeast destination vectors and gene identity of ORFs was ensured by sequencing.

Generation or Source of Entry Constructs; h (human), m (mouse), r (rat).

| **Symbol** | **PCR template** | **Primer pairs (TOPO cloning)**  **FW: forward**  **RV: reverse** | **Clone ID or DNA template for TOPO cloning** |
| --- | --- | --- | --- |
| mPER1 | cDNA (liver) | FW: CAC CAT GAG TGG TCC CCT AGA AGG  RV: CTA GCT GGT GCT GTT TTC TTC | -- |
| mPER2 | cDNA (liver) | FW: CAC CAT GAA TGG ATA CGT GGA CTT CTC  RV: TTA CGT CTG GGC CTC TAT CC | -- |
| mPER3 | pcDNA3HA | FW: CAC CAT GGA TCC CTG TGG AGA CCC  RV: TCA ACT GGT GTC TTC TGC TGG | Kindly donated by  Dr. K. Vanselow |
| mCRY1 | cDNA (liver) | FW: CAC CAT GGG GGT GAA CGC CGT GC  RV: TCA GTT ACT GCT CTG CCG CTG | -- |
| hNONO | -- | -- | RZPDo834C104D/RZPD |
| hWDR5 | -- | -- | IOH4895/RZPD |
| mTIM  (TIMELESS) | cDNA (liver) | FW: CAC CAT GGA CTT GTA CAT GAT GAA CTG  RV: TCA GTC ATC CTC ATC CTC AAT | -- |
| mKIAA1737  (CIPC) | cDNA (liver) | FW: CAC CAT GGA GAG GAA AAT CCC ATC CA  RV: CTA TAC GTC TGG GTG ATC AGA C |  |
| hBHLHB2  (DEC1) | -- | -- | CCSB_14984/MGC |
| hBHLHB3  (DEC2) | cDNA (HEK293) | FW: CAC CAT GGA CGA AGG AAT TCC TCA  RV: TCA GGG AGC TTC CTT TCC TG | -- |
| mCLOCK | -- | FW: CAC CAT GGT GTT TAC CGT AAG CTG TA  RV: CTA CTG TGG CTG GAC CTT GG | -- |
| hNPAS2 | -- | -- | IOH29403/RZPD |
| mBMAL1  (ARNTL) | cDNA (liver) | FW: CAC CAT GGC GGA CCA GAG AAT GGA  RV: CTA CAG CGG CCA TGG CAA G | -- |
| hBMAL2  (ARNTL2) | ARNTL2/pcDNA3 | FW: CAC CAT GGC GGC GGA AGA GGA GG  RV: CTA GAG GGT CCA CTG GAT GT | Kindly donated by  Dr. Nomura  (Ikeda et al., 2000) |
| hNR1D1  (REV-ERBA) | -- | -- | ORF_SEQ_3F10 |
| hNR1D2  (REV-ERBB) | -- | -- | ORF_SEQ_6C06/1-22 |
| hRORA | -- | -- | CCSB_4928/MGC |
| hRORB | -- | -- | IOH39668/RZPD |
| hRORC | -- | -- | CCSB_8508/MGC |
| hDBP | cDNA (HEK293) | FW: CAC CAT GGC GCG GCC TGT GAG C  RV: TCA CAG GGC CCC GTG CTG | -- |
| hNFIL3 | -- | -- | RZPDo839B0796/RZPD |
| hEZH2 | -- | -- | CCSB_143/MGC |
| hRASD1 | -- | -- | CCSB_1134/MGC |
| mCSNK1E | cDNA (liver) | FW: CAC CAT GGA GTT GCG TGT GGG AAA  RV: TCA TTT CCC AAG ATG GTC AAA TG | -- |
| hCSNK1D | cDNA (liver) | FW: CAC CAT GGA GCT GAG GGT CGG GA  RV: TCA TCG GTG CAC GAC AGA CTG | -- |
| mGSK3B | cDNA (liver) | FW: CAC CAT GTC GGG GCG ACC GAG AAC C  RV: TCA GGT GGA GTT GGA AGCTGA | -- |
| hCSNK2A1 | -- | -- | RZPDo839B0979/RZPD |
| hCSNK2A2 | -- | -- | IOH6369/RZPD |
| hCSNK2B | -- | -- | RZPDo834F0628D/SMP |
| hPRKCA | -- | -- | IOH29644/SMP |
| hPRKACA | -- | -- | IOH26286/SMP |
| hPPP1CA | -- | -- | CCSB_3788/MGC |
| hPPP1CB | -- | -- | CCSB_4085/MGC |
| hPPP1CC | -- | -- | CCSB_7076/MGC |
| hPPP2CA | -- | -- | RZPDo834A1014D/RZPD |
| hPPP2CB | -- | -- | RZPDo834A1014D/RZPD |
| hPPP2R1A | -- | -- | RZPDo834A1014D/RZPD |
| hPPP2R1B | -- | -- | RZPDo834A1014D/RZPD |
| hPPP2R5D | -- | -- | RZPDo834A1014D/RZPD |
| hPPP2R5E | -- | -- | RZPDo834A1014D/RZPD |
| rPPP5C | EGDP-PP5 | FW: CAC CAT GGC GAT GGC GGA GGG C  RV: TCA CAT CAT TCC TAG CTG CAG | Kindly donated by Dr. Nkagama  (Fukuda et al., 2007) |
| mBTRC | cDNA (liver) | FW: cac cat gga ccc ggc aga ggc ggt g  RV: tct gga gat gta ggt gta tgt ccg | -- |
| mFBXW11  (BTRC2) | cDNA (liver) | FW: atg gag ccc gac tcg gtg  RV: tct gga gat ata ggt gta gg | -- |
| hFBXL3 | -- | -- | CCSB_14644/MGC |
| hFBXL15  (homolog *Jetlag*) | cDNA (HEK293) | FW: CAC CAT GGA GCA ACC GAT GGA GC  RV: TCA GAC CTG CAG GTT GAC AA | -- |

**1.3 Automated Y2H Screening (referring to Figure 1A**-**C; Figure S1)**

The *L40ccαMATα* yeast strain was independently transformed with prey plasmids encoding the *Gal4* transcription activation domain (N-terminal fusions) while the bait plasmids containing the *LexA* DNA binding domain hybrids (N-terminal fusions) were introduced into the L40ccU *MAT*a strain, respectively. All constructs were tested for auto-activation of the three reporters (*his3*, *ura3* and *lacZ*) by co-transformation of baits or preys with constructs harboring only the transcription activation or DNA binding domain in four independent repetitions in 96 wells. 13% (6 baits) of the constructs have strongly activated the reporters by themselves and were excluded from the Y2H matrix screen (see Figure 1A, Figure S1). For each of the six independent interaction matings, 50 µl of the liquid cultures of the *MAT*a yeast strain were placed into 384-micro titer plates by a pipetting robot (Biomek FX) while the prey colonies were stirred from solid selective media into the liquid cultures using a spotting robot (KBiosystems). The yeast mixtures were then spotted onto YPD (yeast complete medium) agar plates and incubated for at least three days at 30°C. After the mating procedure, colonies were automatically transferred into 348 wells containing SDII liquid (-Leu, -Trp) selective medium and transferred to SDII agar for selection of diploid yeast followed by incubation at 30°C for at least two days. Diploid yeasts were again stirred into liquid and subsequently spotted on solid selective SDIV agar plates (-Leu, -Trp, -Ura, -His) as well as nylon membranes placed on SDIV agar plates. After 6 days at 30°C β-Galactosidase assays were performed with the colonies that grew on membrane. Digital images were taken. Growth and β-Galactosidase activity was analyzed using the Visual Grid (GPC Biotech) software [10,11]. Because of a very low mating efficiency of mPER2 (prey configuration) and mCRY2 (bait configuration) matings with yeast expressing all 45 components were individually performed for these two candidates in 96 well format in six independent experiments. In case that interaction of two components occurred in both the bait and prey configuration the conformation with the highest interaction score was selected for representation. Yeast strains expressing BHLHB2 (DEC1) could not successfully mate with strains expressing BTRC, FBXW11, NPAS2 and PRKCA. In addition, yeast strains expressing PRKCA were not mating with BHLHB2 or PPP2R1B expressing strains.

**1.4 Interactions Detected in Yeast (referring to Table S1)**

The ratio of positive colony growth on SDIV or positive colonies for β-Galactosidase activity and the corresponding mating controls on SDII media was calculated and represented in percent. For example, Clock and Bmal1 were six times positive for interaction on SDIV media underlying six successful matings for diploid yeast on SDII (6 x SDIV positive/6 x SDII positive x 100 = 100% - all of tested interactions have been detected). The analogues calculation was performed for β-Galactosidase activity. PER3, TIMELESS, NR1D1, PRKCA, PPP5C, BTRC and FBXL3 showed no interactions in yeast cells.

**2. Validation of New CLOCK and BMAL1 Interactions in Mammalian Cells**

**2.1 Co-Immunoprecipitation (co-IP) Experiments in HEK293 cells (referring to Figure 1D; Figure S2B**-**D)**

HEK293 cells were lentivirally transduced with constructs expressing Clock-or Bmal1 luciferase fusion proteins in the pLenti6 backbone (Invitrogen). For details of virus production see [4,12]. Cells stably expressing luciferase fusions were transiently transfected with N-terminal MYC-tagged components (pc-myc-CMV-D12) using FuGene 6 (Roche) or Lipofectamine 2000 (Invitrogen) according to the manufacturer’s protocol. 48 hours after transfection cells were harvested in co-IP buffer (20 mM Tris-HCl at pH 8.0; 140 mM NaCl; 1.5 mM MgCl_2_; 1 mM TCEP; 1% Triton-X-100; 10% glycerin) containing a protease inhibitor cocktail (P-8340, Sigma-Aldrich). Lysates with one million luciferase counts were subjected for co-IP experiments. Input counts of 5 µl lysate were detected with the Beta Scout (PerkinElmer) device using 25 µl of LARI (Promega) as a substrate containing reagent for 10 s. Pull-downs were performed with 2 µg of an anti-MYC (NB600-335, Novous Biologicals) or an isoform specific ideotypic (PPP500P, Acris Antibodies) antibody with G PLUS-agarose beads (Santa Cruz Biotechnology) *via* overnight incubation at 4°C and constant agitation. Beads were washed three times in 250 µl washing buffer (20 mM Tris-HCl at pH 8.0; 150 mM NaCl; 0.5% Igepal CA-630). Luciferase activity of beads pellets was measured as performed for input detection. Beta Scout background values were subtracted for all conditions. Validation experiments were performed at least twice with comparable results.

**2.2 Input Detection of MYC-Tagged Components (referring to Figure 1D; Figure S2C)**

Lysates used for co-IP experiments containing MYC-tagged proteins were denatured in SDS-loading buffer (Invitrogen) for 10 min at 95°C. Separation was performed by SDS-PAGE with 4%-12% Bis-Tris gels (Invitrogen). Proteins were transferred to nitrocellulose membrane and incubated with an anti-MYC antibody (NB600-335, Novous Biologicals) according to the manufacturer’s protocol. Membranes were probed with a HRP-conjugated secondary antibody (Santa Cruz Biotechnologies) and chemiluminescence reaction was performed with Super SignalWest Pico substrate (Pierce). Protein bands were visualized using the ChemoCam (Intas) detection system.

**3. Genetic Perturbation Studies in Oscillating Cells**

**3.1 RNAi-Mediated Gene Silencing (referring to Figure 3 and Figure 5A)**

Lentiviruses delivering RNAi (pGIPZ) constructs (Open Biosystems, Huntsville AL) were produced in HEK293T cells in a 96 well plate format essentially as described (Brown et al., 2008). Virus containing supernatants were filtered using Acro-Prep filterplates (Milipore). U2OS reporter cells (with a stably integrated 0.9 kb *Bmal1* promoter fragment) [4,7] were transduced with 100 μl virus filtrate plus 8 µg/μl protamine sulfate (Sigma, Munich, Germany) in white 96-well plates (Nunc, Langenselbold, Germany). After one day, medium was exchanged to puromycin (10 μg/ml) containing medium. Three days later, cells were synchronized with dexamethasone (1 μM) for 30 min. Bioluminescence was recorded for 5 to 7 days in a TopCount luminometer (PerkinElmer) with a stacker unit (sampling rate: ~30 min). RNAi data for 88 network neighborhood genes (Figure 3 and Table S2) are part of a genome-wide screen (n = 1). RNAi experiments for clock core and regulatory components (45 genes; no RNAi construct was available for DEC1 in our library) were performed in three independent experiments (independent virus productions) with 20 non-silencing pGIPZ constructs per 96 well plate serving as controls.

**3.2 Overexpression (referring to Figure 5B)**

U2OS reporter cells were lentivirally transduced with 46 ORFs in pLenti6 backbone (Invitrogen). After ten days of positive selection with blasticidine (10 µg/ml) (Invitrogen) bioluminescence was recorded as described above (3.1). PRKACA overexpression caused cell lethality. Experiments were independently performed three times. Cells expressing GFP (pLenti6) (n = 10 per plate) were considered as controls.

**3.3 Data Analysis and Phenotypic Score (referring to Figure 3 and Figure 5)**

*Network Neighborhood (88 genes)*: period values were determined using an in-house programmed software (ChronoStar), which basically performs a detrending by division of individuals data 24-h-running average and gives parameter estimations by fitting of a cosine wave function: $y=a\cdot\exp(b\cdot t)\cdot\cos(2\cdot\pi\cdot t\cdot24/c+d)$

where *a* = amplitude, *b* = damping, *c* = period and *d* = phase. The reliability of fitted parameters was determined by the correlation coefficient (cc). This software allows batch-processing of large quantities of circadian bioluminescence datasets. Curve fits with cc-values < 0.86 where considered as not reliable and marked for visual inspection. Those datasets which could not be described properly by above cosine function where excluded from further analysis. Datasets with cc < 0.86 and an arrhythmic characteristic where labeled as arrhythmic. Individual period values where normalized on global period effects of lentiviral load as determined by GFP fluorescence (Infinite F200pro, Tecan, Austria) of U2OS cells right before bioluminescence recording, as well as on optimized plate mean value (arbitrarily set to 24 hours). This optimized mean value was determined by an iterative process, where extreme values (more than two standard deviations away from the respective actual mean) were excluded. We considered only those values as altered phenotypes, which deviated at least 0.5 hours from an optimized mean value of the respective plate. We evaluated 88 genes from circadian network neighborhood. For 82 genes we found at least one targeting construct in our pGIPZ library (this was not the case for the genes: CEBPA, DDIT3, EP300, HSP90AA1, NOS1 and PXN). Genes from the network neighborhood, which showed at least two RNAi constructs with similar period phenotype, *i.e.* extreme period changes in the same direction or one period phenotype plus one arrhythmic phenotype, where considered as neighborhood hits (see also Table S2).

*Clock Core and Regulatory Components (46 genes)*: data were analyzed as described above. Period differences to control values (mean of 20 non-silencing controls/plate for knockdowns; mean of 10 GFP expressing controls/plate for overexpression) within individual plates of > 0.5 h were considered as altered phenotypes. Amplitude and damping values that were more than 2.5 standard deviations different from the mean of the controls were considered as significant. Time series with a bad fit to a cosine function, i.e. a correlation coefficient cc < 0.8 for knockdowns and cc < 0.9 for overexpression were classified as arrhythmic. Arrhythmic phenotypes were confirmed or rejected by visual inspection of the corresponding raw data.

*Phenotypic score*: Oscillations classified as arrhythmic based on bad fit to a cosine function (see above) were scored with 4 points. Period between 0.5 h and 1 h different from controls, between 1 h and 2 h, and more than 2 h were scored with 1, 2 or 3 points, respectively. Low amplitude and high damping were scored with 1 point and not assigned if a phenotype was already classified as arrhythmic. Phenotypic scores represent the sum of points from knockdown and overexpression experiments for an individual component in all categories (see Figure 5A,B; Figure S4 and Table S4).

**4. CLOCK/BMAL1 Co-Transactivation Assay (referring to Figure 7A,B; Figure S7A,B)**

An artificial 6 *E-box* luciferase reporter (pGL3, Promega), Clock/Bmal1 (pDEST26, Invitrogen) and individually all discovered CLOCK and BMAL1 interactos and their paraloges (pLenti6, Invitrogen) that have been found by high-throughput interaction mapping in yeast were transiently transfected with Lipofectamine 2000 (Invitrogen) according to the manufacturer’s protocol in HEK293 cells (see also Table S1). CLOCK/BMAL1 co-transactivation assays were performed as previously described [13,14]. Normalization was performed to Renilla-luciferase signals in lysates. Equal DNA amounts in transfections were ensured by the addition of *lacZ* DNA in the corresponding backbone. Signal detection was performed with the Dual-Luciferase Reporter Assay (Promega) in the Orion II Luminometer plate reader (Berthold Detection Systems). Experiments with all candidates were independently performed three times. A fourth repetition was only performed with candidates that showed consistent results in the previous repetitions (n = 3). Regulatory and scaffolding subunits were co-transfected with their corresponding catalytic subunits to simulate functional holoenzymes. Overexpression of candidates had no effect on the reporter alone (data not shown).

**5. Co-Immunoprecipitation (Co-IP) with Endogenous Components from Mouse Liver (referring to Figure 7C)**

Animals were sacrificed at circadian time points (CT) CT12 or CT0 for liver preparation. Whole-cell lysates were generated and co-IPs were performed as described under 2.1. Co-IPs and Western blot analysis were performed with an anti-BMAL1antiboby (kindly donated by the M. Brunner laboratory; specificity was intensively characterized using *Bmal1* knock out mouse tissue [15] or an anti-PPP1Cα antibody (sc-7482, Santa Cruz Biotechnology). Controls were performed with the following ideotypic antibodies (sc-3878, sc-2037 Santa Cruz Biotechnology). 100 µg of total lysate were loaded as input controls. 500 µg of protein were subjected for immunoprecipitation experiments.

**6. BMAL1 Stability Measurements**

**6.1 Protein Stability Measurement of EGFP-BMAL1 (referring to Figure 7D; Figure S7B, C)**

U2OS cells stably carrying the fluorescence reporter (see Figure 7D left) as described in [16,17] either with BMAL1, CLOCK or a EGFP control (d4EGFP) fusion proteins were transduced with lentiviral supernatant of PP1Cα or GSK3β (pLenti6, Invitrogen) including 8 µg/ml protamine sulfate (Sigma-Aldrich). After positive selection for 10 days with blasticidine (10 µg/mL, Invitrogen) 1 x 10^5^ cells were seeded into 24-well plates. After 3 - 4 days, cells were re-suspend in 50 µl 1xPBS + 0.5 % FBS (PAA) + 0.1 % NaN_3_ (Serva) and fluorescence was red-out using FACS Canto II (Becton Dickinson) flow cytometry instrument. Data were analyzed using FCS Express 4 Flow Research Edition (DeNovo Software). Raw data of red fluorescence of DsRed and green fluorescence of EGFP fluorescence intensities of DsRed positive cells were exported to Excel (Microsoft 2010). Autofluorescence intensities of FITC and PE negative U2OS cells were subtracted and the ratio EGFP/DsRed of each cell was calculated. A curve fit using Origin7 software (Lorentzian fit, OriginLab) was applied on the distribution of ratios of analyzed cells. Zero point of the slope (= maxima of the distribution where most cells display a certain ratio) was assigned as the protein stability index (PSI). High PSI values refer to high green fluorescence intensities, thus high abundant fusion proteins reflecting high protein stability and *vice versa*.

**6.2 Endogenous BMAL1 Levels in the Presence of PPP1C**α **(referring to Figure 7E,F)**

*Steady-State*: U2OS *Bmal1-*promoter luciferase reporter cells stably expressing PPP1Cα or GFP (pLenti 6, Invitrogen) were harvested in RIPA buffer (1 % Igepal CA-630, 0.5 % sodium-deoxycholat, 0.1 % SDS in 1 x PBS) containing protease inhibitor cocktail (P-8340, Sigma-Aldrich). 30 µg of total lysate was loaded for Western blot analysis as performed in 2.1 using the anti-BMAL1 antibody from M. Brunner’s laboratory. Membranes were probed with an anti-Actin IgG (A-5441, Sigma-Aldrich) as loading controls. Quantification was performed with ImageJ 1.44p software (National Institutes of Health).

*Cycloheximide* (CHX) *Time Series*: U2OS cells as described above were treated with CHX (C4859, Sigma-Aldrich) using a 0.71 mM final concentration. Cells were harvested at the following selected time points: 3 h, 4 h, 5 h and 6 h. Western blotting and incubation with antibodies were performed as described above.

**6.3 Detection of Overexpression Efficiency via Quantitative PCR (q-PCR) (referring to Figure S7D)**

Stable overexpression of PPP1Cα was tested in U2OS *Bmal1-*promoter luciferase reporter cells. RNA was generated with the RNeasy Mini Kit (Qiagen) and reversely transcribed to cDNA using Superscript RNase H^-^ (Invitrogen). SYBRGreen fluorescence assays using human QuantiTect Primer Assays (Qiagen) were performed. Gene expression was monitored by an ABI PRISM 7000 detection system (Applied Biosystems). Transcript levels were normalized to Gapdh as internal sample controls and assessed by the 2^-ddCt^ method as described by [4].

**7. Network Visualization (referring to Figure 1, Figure 2, Figure 6 and Figure S6)**

Network was depicted using the Cytoscape 2.8.1 software as available at [www.cytoscape.org](http://www.cytoscape.org)

**II. Bioinformatics Analysis**

**1. Construction of Protein-Protein Interaction Networks (referring to Figure 2 and Figure S3A)**

For network analyses, proteins were indexed by corresponding human Entrez Gene IDs. For a proteome-wide interaction network, data curated in the original databases HPRD (binary interactions only), DIP, BIND, BIOGRID and INTACT as stored in the UNIHI4 version were integrated [18]. This generated a global network of 9982 proteins and 45775 interactions. The network was used to identify additional interactions between the proteins in the circadian network (PPIs among 46 components (clock core and regulatory components)) as well as to assess the molecular context of the circadian network. First, the global protein networks were examined for overlap (common proteins and interactions) with the circadian PPI network. Identified additional interactions between the 46 components were added to enlarge the circadian PPI network. Furthermore, we included proteins interactions based on a literature review that we conducted (see also Table S1).

**2. Extension of the Circadian Protein-Protein Interaction Network (referring to Figure 2 and Figure S3A)**

First, all direct interaction partners of 24 clock core components (without regulatory components) were identified in the global protein network. In total, these resulted in 88 interaction partners. Second, we searched for additional interactions between any of the 46 proteins (clock core and regulatory components) and 88 proteins in the neighborhood in the global network. Including these additional interactions lead to an extended network of 134 proteins and 625 interactions (see also Table S1).

**3. Determination of Periodic Expression (referring to Figure 2, Figure 6C and Figure S6A)**

To identify periodic expression patterns, the data set by Hughes et al. [19] measuring gene expression in mouse liver was utilized. The microarray dataset includes consecutive sampling over 48 hours (with intervals of 1 hour) for 45101 probe sets. These probe sets correspond to 21308 unique mouse Entrez Gene identifiers. For derivation of probe set intensities, the RMA method as implemented in the Bioconductor *affy* package was utilized.

To evaluate the periodicity, the expression vectors mRNA profiles were standardized and subsequently corresponding Fourier scores for a periodicity of 24 h were calculated. The Fourier score is defined as

$$F\left[x\right]=\sqrt{\sum_{i}\left(\cos\left(2*\pi*t_{i}/\mathrm{T}\right)*x_{i}\right)^{2}+\sum_{i}\left(\sin\left(2*\pi*t_{i}/\mathrm{T}\right)*x_{i}\right)^{2}}$$

where ***x*** is the standardized expression vector (mean(***x***)=0; sd(***x***) =1) for the gene, *T* is the period (in our case 24 h), and *x_i_* is the measured expression at time point *t_i_*. Statistical significance (*i.e.* false discovery rate (FDR)) was derived through comparison using randomly permutated time series [20]. The expression analysis was performed using the Bioconductor *cycle* package.

**4. Interactions and Expression of Regulatory Proteins (referring to results section)**

To compare the number of interactions of regulatory and non-regulatory proteins in the human interactome, we first calculated the number of interactions for each protein in the global protein-protein interaction networks derived from UniHI database. For the classification of regulatory proteins, we utilized gene annotation from the GO database. Proteins whose corresponding gene were associated with the GO category `Regulation of biological process´ (GO:0050789) were assigned as regulatory proteins (N = 4635). The remaining proteins were classified as non-regulatory (N = 5347). Subsequently, the numbers of interactions of proteins in both groups were compared. We observed that regulatory proteins tend to have more interactions than non-regulatory proteins. The mean number of interactions for regulatory proteins was 12.74, whereas non-regulatory proteins have in average only 5.64 interactions. Statistical testing demonstrated that this difference is highly significant (Wilcoxon test: p < 10^-15^).

We also examined the relationship between periodic expression and number of interactions. First, we calculated the number of interactions for human proteins for which the mouse ortholog is showing significant periodic expression (FDR < 0.01; N = 2468). The average number was 11.31 interactions compared to 8.81 for proteins with a mouse orthologs that is not periodically expressed (N = 5011). The difference is statistically highly significant (Wilcoxon Rank test; p < 10^-10^) indicating that proteins with periodic expression tend to have more interactions compared to constitutively expressed proteins. Secondly, we examine whether the reverse is valid *i.e.* interaction-rich proteins tend to be periodically expressed. To this end, we classified proteins with more than 50 interactions as interaction-rich (N = 762), and compared their periodic expression with proteins that have less than 5 interaction (N = 6654). Notably, almost 42% of the interaction-rich proteins were periodically expressed compared to only 29% for interaction poor. Statistical evaluation of the difference by Chi-squared test indicated a high significance (p < 10^-5^).

**5.** **Identification of Dynamic/Rhythmic Protein-Protein Interactions (referring to Figure 4B, Figure 6D and Figure S6C)**

To obtain an approximation of the dynamics of PPI in the network, we assumed that the abundance *A_C_* of the complex *C* formed by two interacting proteins P_1,2_ through a PPI is proportional to the expression *E* of P_1,2_. Thus, the abundance *A_C_(t)* over time can be approximated by the product of expression vectors mRNA profiles *E*_P1_(t) * *E*_P2_(t) which was then associated with the corresponding PPI between P_1_ and P_2_. As proxy for the expression, the transcript levels over time were utilized, thus

$$PPI_{12}\sim A_{c}(t)\approx c*A_{P1}(t)*A_{P2}(t)\to A_{c}(t)\approx E_{P1}(t)*E_{P2}(t)$$

The statistical significance of the product of expression vectors mRNA profiles was calculated in the same manner as for simple expression vectors (*i.e*. using the Fourier-score and permutated time series as background model) after standardization (*i.e.* mean (*E_P_*_1_ * *E_P_*_2_) = 0; sd (*E_P_*_1_ * *E_P_*_2_) = 1). Additionally, a phase was assigned to a periodic interaction through shifting a cosine (with periodicity 24 h) along the time axis and measuring the overlap of the expression levels with the cosine curve. PER1-3 mRNA profiles were shifted + 6 hours (see [21]). The time shift leading to a maximum overlap was considered as the phase α of the PPI and ranges from 0 to 24 h (see also Table S3).

**6. Characterization of Protein Hubs in Network (referring to Figure S5)**

To examine the role of hubs in the circadian network, we analyzed the co-expression of interacting proteins. For all proteins in the network, we calculated the average of Pearson correlation coefficient (PCC) of the expression vectors for the protein and its interacting partners. To estimate the significance of the calculated PCCs, they were compared to average PCCs obtained for randomly selected interaction pairs. As the average PCC depends on the number of interaction partners, independent background distribution were repeatedly generated for each protein in the network. Importantly, the number of interactions was conserved in the background distribution. Two methods for the generation of the background distributions were used: 1) Random pairs (N = 10^4^) were drawn from the full set of genes included in the expression data set (`random interaction pairs´) and their PCC were calculated. For the calculation of the average PCC, the number of interaction pairs, which were generated, was the same number as observed for a chosen protein in the circadian network. 2) For each protein to be assessed only interaction partners were randomly drawn from the genes included in the expression data set and the PCC calculated (`random interaction partner´). In this way, the expression profile of the protein in the network is included in the generation of the background distribution of average PCC and the influence of potential global patterns on the calculation of PCC is conserved. Again, the number of drawn interaction partners was the same as observed for the circadian network (see also Table S3).

For the calculation of the statistical significance, p-values were obtained by comparing the average PCCs from the background distribution to the observed one. P-values for two cases were calculated for the occurrence of: a) the observed average PCC is larger than expected based on the background distribution; b) the observed average PCC is smaller than expected based on the background distribution. Finally, all derived p-values were adjusted using the Benjamini-Hochberg method.

In general, both approaches (using on random interaction pairs or random interaction partners) produced similar significance values (Spearman correlation ~ 0.95). Some examples for the observed average PCC and the background distributions are shown in (Figure S5).

**7. Dynamic Interactions Between Biological Processes in the Circadian Network (referring to Figure 6A)**

To examine the dynamic coupling between biological processes in the circadian network, we first selected the processes which were examined in the analysis. Biological processes (BP) and their associated genes (or respectively proteins) were given by the annotation in Gene Ontology ([www.geneontology.org](http://www.geneontology.org)). Here, we chose processes for which associated proteins were overrepresented in the circadian network. For calculation of statistical significance of enrichment, hypergeometric test (which is equivalent to Fisher´s exact test) with subsequent Benjamini-Hochberg for multiple testing was utilized. A FDR cut-off of 0.25 for significance was set.

A well-known difficulty in the interpretation of enrichment analysis for Gene Ontology categories is occurrence of highly overlapping terms. This is caused by the hierarchical structure of Gene Ontology: significance of enrichment in children terms frequently result in significance of their parent terms *(e.g*. significance in proteins linked to the term `Intracellular signal transduction´ contribute to the statistical enrichment of proteins in the term `Signal transduction´). To reduce the set of significant terms to a smaller and less redundant set of terms, we used following filtering procedure: Categories were excluded if the majority of included genes (*i.e.* more than 50%) had been associated with a single category having less genes. This procedure generally led a reduced set of more specific categories facilitating inspection of results. Applied to the 134 genes included in circadian network and the full set of categories for biological processes derived from Gene Ontology through the Bioconductor GO package, 16 significant categories with a minimum size of 10 associated genes were derived (see Table S5).

Next, we counted the number of interactions between proteins for all possible pairs of processes. Also, the number of dynamic interactions (with a significance of periodicity of 10^‑5^) was counted. Using the calculated numbers, we tested whether dynamic interactions were over-represented in the total set of interactions between a pair of categories using Fisher`s exact test with subsequent Benjamini-Hochberg adjustment for multiple testing. A network of biological processes was then constructed based on the significance for the enrichment in dynamic interactions between categories.

For the reduced set of GO categories, setting a threshold for significance of FDR < 0.1 resulted in a network with 12 links between 11 processes. The central position is constituted by the BP category `Circadian rhythm´ which is linked to the 10 other categories by dynamic interactions (see Figure 6A, Table S5).

**8.** **KEGG Pathway Analysis of Components of the Network Neighborhood** **(referring to Figure 6B)**

The circadian network neighborhood was examined for enriched KEGG pathways. To this end, the 88 proteins in the neighborhood were annotated with pathway information derived from KEGG through the Bioconductor KEGG package. The significance for enrichment was calculated using the hypergeometric test which is equivalent to the Fisher´s exact test. Pathways with less than 5 associated proteins from the network neighborhood were excluded. For multiple testing, an adjustment by the Benjamini-Hochberg method was conducted and FDR were derived. A threshold of FDR < 0.25 was applied. The significant pathways, the number of associated proteins and their significance are displayed in Figure 6B (see also Table S5).

**9. Overlap with Reference Gene Sets**

Significance was calculated using the hypergeometric test (which is equivalent to Fisher´s exact test) with the complete lists of genes included in the global network as reference set.

The overlap with the cancer census list ([www.sanger.ac.uk/genetics/CGP/Census](http://www.sanger.ac.uk/genetics/CGP/Census)) of 427 genes is:

- for the clock core and regulatory components (46 proteins): EZH2, PER1, NONO

– 3 genes – p = 0.10

- for the full extended network (134 proteins):

PER1, NONO, EZH2, EP300, CREBBP, HSP90AA1, RARA, HLF, NCOA2, NCOA1, SFPQ, MEN1, MLL, MYC, AKT1, PML, SUZ12, ATF1, DDIT3, CEBPA

**–** 20 genes – p = 2.8 * 10^-8^

- for the neighborhood only (88 proteins): EP300, CREBBP, HSP90AA1, RARA, HLF, NCOA2, NCOA1, SFPQ, MEN1, MLL, MYC, AKT1, PML, SUZ12, ATF1, DDIT3, CEBPA

– 17 genes – p = 3.8 * 10^-9^

**10. Identification of Structural Modules (referring to Figure 6C and Figure S6A)**

In order to identify dense local sub-networks (*i.e*. structural modules), two algorithms were used: 1. the MCODE algorithm (baderlab.org/Software/MCODE) and 2. the ClusterOne algorithm ([www.paccanarolab.org/cluster-one](http://www.paccanarolab.org/cluster-one)). For both algorithms, their Cytoscape implementation was utilized. The two methods are similar in their general procedure, *i.e.* to detect first dense local networks and them to expand them.

Significance of periodicity of interaction, is represented by edge color with red representing highly significant (FDR = 10^-5^) and yellow/white representing low significance of periodic expression. Similarly, edge width represents also the significance of periodicity of interaction with larger width representing higher significance. Yellow circles highlight rhythmic RNA profiles; circle width corresponds to significance of periodic expression (see Figure 6C, Figure S6A). Calculation of significance of functional enrichment was based on Fisher´s exact test with Benjamini-Hochberg adjustment using the annotation provided by the GO Bioconductor package.

**10.1 MCODE Modules**

To identify structural modules using the MCODE algorithm, the node score cutoff parameter was set to 0.05. For other parameter, the default values (Version 1.32) were used. This parameter setting led to small structural modules facilitating their inspection. In total, 9 clusters were identified, 5 of which were simple triangles and disregarded in the subsequent analysis. The top 4 modules are as follows:

1. **Module (MCODE)**


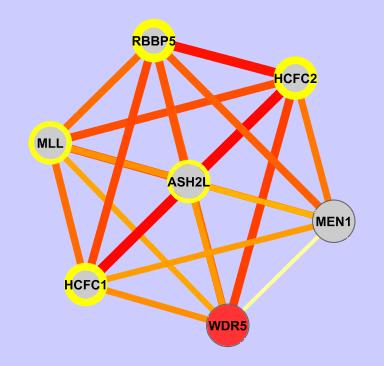


This module obtained rank 1 and a score of 3. (The score produced by MCODE is the average number of distinct interactions per protein). It includes 7 proteins and 21 interactions. 5 proteins are significantly (FDR < 0.01) periodically expressed. The GO terms *histone methyltransferase complex* (FDR < 10^-11^; 5 genes: HCFC1, MEN1, MLL, RBBP5, ASH2L) and *transcription from RNA polymerase II promoter* (FDR = 0.0008; 5 genes: HCFC1, MEN1, MLL, ASH2L, HCFC2) are significantly enriched.

1. **Module (MCODE)**

**
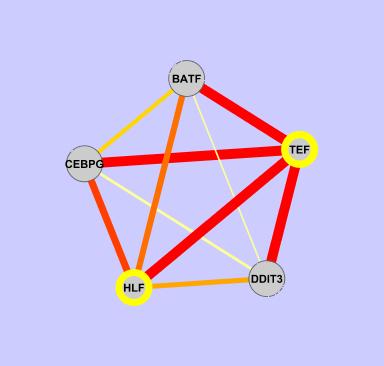
**

This module obtained rank 2 and a score of 2. It includes 5 proteins and 10 interactions. The GO term *transcription from RNA polymerase II promoter* (FDR= 0.004; 4 genes: CEBPG, DDIT3, HLF, TEF) as well as the Pfam family *Basic region leucine zipper* (FDR < 10^-10^); 4 genes: CEBPG, DDIT3, HLF, TEF) are significantly enriched.

1. **Module (MCODE)**


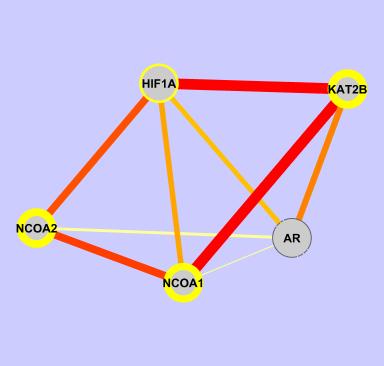


This module obtained rank 3 and a score of 1.8. It includes 5 proteins and 9 interactions. The GO terms *transcription coactivator activity* (FDR = 0.0016; 3 genes: NCOA1, KAT2B, NCOA2) *and positive regulation of transcription from RNA polymerase II promoter* (FDR = 0.0004; 4 genes: AR, HIF1A, NCOA1, NCOA2) are significantly enriched*.*

1. **Module (MCODE)**


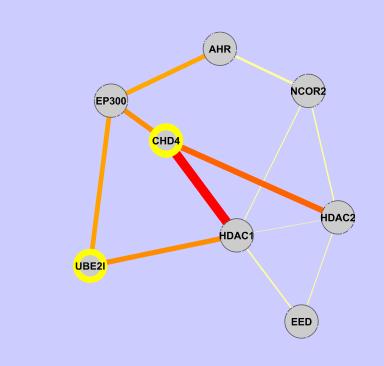


This module obtained rank 4 and a score of 1.5. It includes 8 proteins and 12 interactions. The GO terms *chromatin modification* (FDR < 10^-4^; 5 genes: CHD4, EP300, HDAC1, HDAC2, EED), *transcription factor binding* (FDR < 10^-4^; 6 genes: AHR, CHD4, EP300, HDAC, HDAC2, NCOR2), *negative regulation of transcription* (FDR < 10^-3^; 4 genes: HDAC2, UBE2I, EED, NCOR2) are significantly enriched.

**10.2 ClusterOne Modules:**

Besides MCODE, we used the ClusterOne Cytoscape plugin to detect structural modules. For the detected modules, the density, quality and p-value of the modules are reported. Density of a structural module is defined by the number of observed interactions within the module divided by the maximum number of possible interactions. The quality of a module is the number of internal interactions divided by the sum of internal interactions and interactions with proteins outside the module. The p-value is based on a one-sided Mann-Whitney U test distinguishing internal interactions and interactions with proteins outside the module. More details are available at [www.cs.rhul.ac.uk/home/tamas/assets/files/cl1/cl1-cytoscape-0.92.html](http://www.cs.rhul.ac.uk/home/tamas/assets/files/cl1/cl1-cytoscape-0.92.html)

1. **Module (ClusterOne)**

**
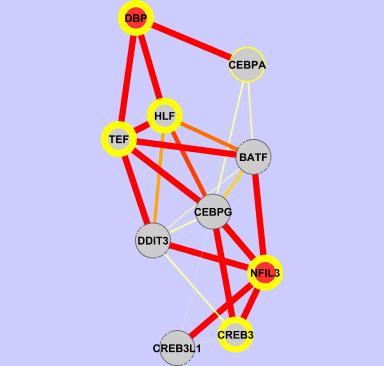
**

This module has 10 proteins connected by 24 interactions. It obtained a density of 0.54, a quality of 0.57 and a p-value of 0.003 by the ClusterOne algorithm. The GO terms *rhythmic process* (FDR < 10^-4^; 4 genes: DBP, HLF, NFIL3, TEF) and *RNA polymerase II transcription factor activity* (FDR < 10^-3^; 4 genes: CEBPA, DBP, TEF, BATF) as well as the Pfam family *Basic region leucine zipper* FDR < 10^-18^; 7 genes: CEBPA, CEBPG, DBP, DDIT3, HLF, NFIL3, TEF) were significantly enriched.

1. **Module (ClusterOne)**

**
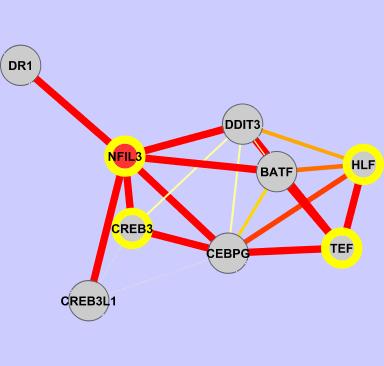
**

This module has 9 proteins connected by 20 interactions. It obtained a density of 0.56, quality of 0.58 and a p-value of 0.004 by the ClusterOne algorithm. The GO terms *transcription from RNA polymerase II promoter* (FDR = 0.00013, 6 genes: CEBPG, DDIT3, DR1, HLF, NFIL3, TEF)*, transcription corepressor activity* (FDR < 10^-3^; 3 genes: DDIT3, DR1, NFIL3) as well as the Pfam family *Basic region leucine zipper* (FDR < 10^-11^; 5 genes CEBPG, DDIT3, HLF, NFIL3, TEF) are significantly enriched.

1. **Module (ClusterOne)**

**
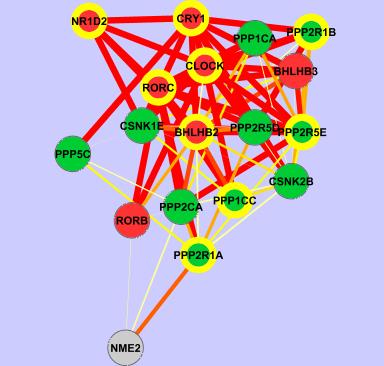
**

This module has 18 proteins connected by 77 interactions. It obtained a density of 0.50, quality of 0.42 and a p-value of 0.01 by the ClusterOne algorithm. The KEGG pathways *Circadian rhythm – mammal* (FDR < 10^-7^; 5 genes: CRY1, CSNK1E, BHLHE40/DEC1, CLOCK, BHLHE41/DEC2) and *Wnt signaling pathway* (FDR < 10^-5^; 7 genes; CSNK1E, CSNK2B, PPP2CA, PPP2R1A, PPP2R1B, PPP2R5D, PPP2R5E) as well as the GO term protein phosphatase type 2A complex (FDR < 10^-5^; 4 genes: PPP2CA, PPP2R1A, PPP2R5D, PPP2R5E) are significantly enriched.

1. **Module (ClusterOne)**

**
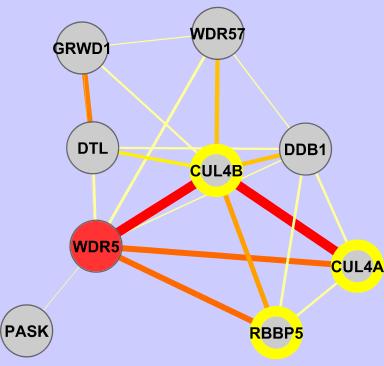
**

This module has 9 proteins connected by 21 interactions. It obtained a density of 0.58, quality of 0.53 and a p-value of 0.015 by the ClusterOne algorithm. The GO terms *response to DNA damage stimulus* (FDR = 0.0017; 4 genes; DDB1, CUL4B, CUL4A, DTL) and *Nucleotide excision repair* (FDR < 10^-5^; 3 genes DDB1, CUL4B, CUL4A) as well as the Pfam family *WD domain, G-beta repeat* (FDR < 10^-6^; 5 genes: WDR5, DTL, RBBP5, GRWD1, SNRNP40) are significantly enriched.

1. **Module (ClusterOne)**

**
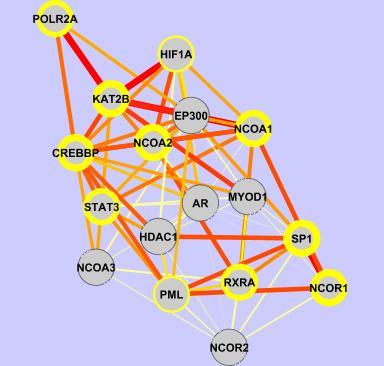
**

This module has 17 proteins connected by 68 interactions. It obtained a density of 0.50, quality of 0.407 and a p-value of 0.015 by the ClusterOne algorithm. The GO terms *histone acetyltransferase activity* (FDR < 10^-8^; 5 genes: CREBBP, EP300, NCOA3, NCOA1, KAT2B*, positive regulation of transcription from RNA polymerase II* promoter (FDR < 10^-8^; 9 genes: AR, EP300, HDAC1, HIF1A, MYOD1, RXRA, SP1, NCOA1, NCOA2) as well as the KEGG pathways *Notch signaling pathway* (FDR < 10^-5^; 5 genes; CREBBP, EP300, HDAC1, KAT2B, NCOR2) ; *Pathways in cancers* (FDR < 10^-4^; 8 genes: AR, CREBBP, EP300, HDAC1, HIF1A, PML, RXRA, STAT3) and *Huntington's disease* (FDR < 10^-3^; 5 genes: CREBBP, EP300, HDAC1, POLR2A, SP1) are significantly enriched.

1. **Module (ClusterOne)**


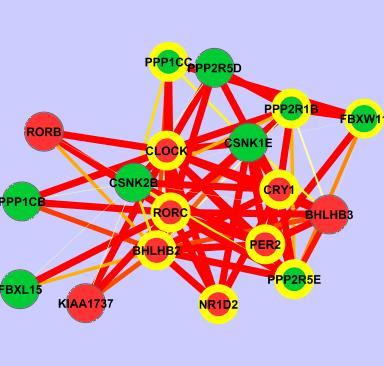


This module has 18 proteins connected by 78 interactions. It obtained a density of 0.51, quality of 0.43 and a p-value of 0.02 by the ClusterOne algorithm. The KEGG pathways *circadian rhythm – mammal* (FDR < 10^-10^; 6 genes: CRY1, CSNK1E, BHLHE40/DEC1, PER2, CLOCK, BHLHB3/DEC2) and *Wnt signaling pathway* (FDR < 10^-4^; 6 genes: CSNK1E, CSNK2B, PPP2R1B, PPP2R5D, PPP2R5E, FBXW11) are significantly enriched.

1. **Module (ClusterOne)**

**
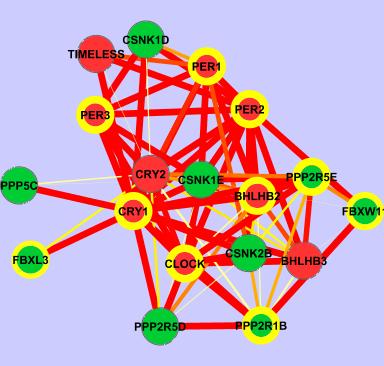
**

This module has 18 proteins connected by 77 interactions. It obtained a density of 0.50, quality of 0.41 and a p-value of 0.025 by the ClusterOne algorithm. The KEGG pathways *circadian rhythm – mammal* (FDR < 10^-21^; 10 genes: CRY1, CRY2, CSNK1D, CSNK1E, PER1, BHLHE40/DEC1, PER3, PER2, CLOCK, BHLHB3/DEC2) and *Wnt signaling pathway* (FDR < 10^-4^; 6 genes CSNK1E, CSNK2B, PPP2R1B, PPP2R5D, PPP2R5E, FBXW11) are significantly enriched.

**11. Dynamic Interactome (referring to Figure 6D; Figure S6B,C)**

To examine circadian coupling between processes on a systems-wide level, all PPIs of the compiled human interactome (N = 45775) were assessed for dynamic behavior. Human proteins were mapped to their orthologs in mouse. As previously for the circadian network, the periodicity of interactions was based on the expression data for mouse liver over the time span of 48 hours. In total, the dynamic behavior of 30413 interactions could be determined. (For the remaining interactions, either corresponding gene expression data was not available or the mouse orthologs could not been found).

For the determination of significance in periodic expression, the described Fourier-based approach with random permutations as background model was used. A threshold of FDR < 10^-5^ was set. This resulted in the identification of 2788 significantly dynamic interactions (see Figure 6D and Figure S6B). The full lists of interactions containing the human proteins and their mouse orthologs, corresponding gene symbols, significance of periodic expression and offset of individual interactions partners as well as of the interaction can be found in Table S5.

For evaluation, which biological processes are connected in a dynamical manner, we performed an enrichment analysis for the 1979 proteins with dynamic interactions. The analysis was based on their GO slim annotation. To obtain non-redundant set of categories for biological processes, the previously described filtering procedure was used *i.e*. categories are excluded if the majority of included genes are associated with a single category having less genes. We obtained 27 biological processes with a significant enrichment of FDR < 0.25. Next, we mapped the dynamic interactions to the corresponding biological processes. As before, the significance of over-representation of dynamical interactions between categories was calculated. Connections which were significantly dynamical enriched were displayed as network (see Figure 6D middle). Transcription and signal transduction were detected to be central hubs in the network of processes coupled by dynamic interactions. In contrast, processes like cellular homeostasis and lipid metabolism tended to be linked to other processes via static interactions. The p-values for enrichment of dynamic interactions between processes can be found in Table S5.

As an alternative approach to examine which biological processes tend to be linked by the dynamic interactions, we compared the wiring of the observed interaction network with randomized versions of it. For randomization, the degree distribution of each protein was conserved. To this end, the interaction between protein pairs were randomly switched *i.e*. interactions between proteins pairs A-B and C-D were changed to A-C and B-D. Every interaction was repeatedly switched in average 100 times. For the randomized networks, the number of dynamic interactions between the biological processes were counted and compared with the observed network. Altogether, 1000 randomized networks were independently generated. We found that for 26 pairs of biological processes, the numbers of dynamic interactions were higher than in any of the randomized network. This number of pairs increased if we relax the requirement. We obtained 52 pairs if we required that the number of dynamic interactions between processes should not be higher in 10 out of the 1000 (see Figure S6C).

**References (Supporting Information and Table S1)**

1. Gallego M, Virshup DM (2007) Post-translational modifications regulate the ticking of the circadian clock. Nat Rev Mol Cell Biol 8: 139-148.

2. Jakubcakova V, Oster H, Tamanini F, Cadenas C, Leitges M, et al. (2007) Light entrainment of the mammalian circadian clock by a PRKCA-dependent posttranslational mechanism. Neuron 54: 831-843.

3. Huang G, Chen S, Li S, Cha J, Long C, et al. (2007) Protein kinase A and casein kinases mediate sequential phosphorylation events in the circadian negative feedback loop. Genes Dev 21: 3283-3295.

4. Maier B, Wendt S, Vanselow JT, Wallach T, Reischl S, et al. (2009) A large-scale functional RNAi screen reveals a role for CK2 in the mammalian circadian clock. Genes Dev 23: 708-718.

5. Busino L, Bassermann F, Maiolica A, Lee C, Nolan PM, et al. (2007) SCFFbxl3 controls the oscillation of the circadian clock by directing the degradation of cryptochrome proteins. Science 316: 900-904.

6. Godinho SI, Maywood ES, Shaw L, Tucci V, Barnard AR, et al. (2007) The after-hours mutant reveals a role for Fbxl3 in determining mammalian circadian period. Science 316: 897-900.

7. Zhang EE, Liu AC, Hirota T, Miraglia LJ, Welch G, et al. (2009) A genome-wide RNAi screen for modifiers of the circadian clock in human cells. Cell 139: 199-210.

8. Koh K, Zheng X, Sehgal A (2006) JETLAG resets the Drosophila circadian clock by promoting light-induced degradation of TIMELESS. Science 312: 1809-1812.

9. Barnes JW, Tischkau SA, Barnes JA, Mitchell JW, Burgoon PW, et al. (2003) Requirement of mammalian Timeless for circadian rhythmicity. Science 302: 439-442.

10. Goehler H, Lalowski M, Stelzl U, Waelter S, Stroedicke M, et al. (2004) A protein interaction network links GIT1, an enhancer of huntingtin aggregation, to Huntington's disease. Mol Cell 15: 853-865.

11. Stelzl U, Worm U, Lalowski M, Haenig C, Brembeck FH, et al. (2005) A human protein-protein interaction network: a resource for annotating the proteome. Cell 122: 957-968.

12. Schmutz I, Wendt S, Schnell A, Kramer A, Mansuy IM, et al. (2011) Protein phosphatase 1 (PP1) is a post-translational regulator of the mammalian circadian clock. PLoS One 6: e21325.

13. Gekakis N, Staknis D, Nguyen HB, Davis FC, Wilsbacher LD, et al. (1998) Role of the CLOCK protein in the mammalian circadian mechanism. Science 280: 1564-1569.

14. Zhao WN, Malinin N, Yang FC, Staknis D, Gekakis N, et al. (2007) CIPC is a mammalian circadian clock protein without invertebrate homologues. Nat Cell Biol 9: 268-275.

15. Rey G, Cesbron F, Rougemont J, Reinke H, Brunner M, et al. (2011) Genome-wide and phase-specific DNA-binding rhythms of BMAL1 control circadian output functions in mouse liver. PLoS Biol 9: e1000595.

16. Yen HC, Elledge SJ (2008) Identification of SCF ubiquitin ligase substrates by global protein stability profiling. Science 322: 923-929.

17. Yen HC, Xu Q, Chou DM, Zhao Z, Elledge SJ (2008) Global protein stability profiling in mammalian cells. Science 322: 918-923.

18. Chaurasia G, Malhotra S, Russ J, Schnoegl S, Hanig C, et al. (2009) UniHI 4: new tools for query, analysis and visualization of the human protein-protein interactome. Nucleic Acids Res 37: D657-660.

19. Hughes ME, DiTacchio L, Hayes KR, Vollmers C, Pulivarthy S, et al. (2009) Harmonics of circadian gene transcription in mammals. PLoS Genet 5: e1000442.

20. Futschik ME, Herzel H (2008) Are we overestimating the number of cell-cycling genes? The impact of background models on time-series analysis. Bioinformatics 24: 1063-1069.

21. Lee C, Etchegaray JP, Cagampang FR, Loudon AS, Reppert SM (2001) Posttranslational mechanisms regulate the mammalian circadian clock. Cell 107: 855-867.

22. Brown SA, Ripperger J, Kadener S, Fleury-Olela F, Vilbois F, et al. (2005) PERIOD1-associated proteins modulate the negative limb of the mammalian circadian oscillator. Science 308: 693-696.

23. Honma S, Kawamoto T, Takagi Y, Fujimoto K, Sato F, et al. (2002) Dec1 and Dec2 are regulators of the mammalian molecular clock. Nature 419: 841-844.

24. Yin L, Wang J, Klein PS, Lazar MA (2006) Nuclear receptor Rev-erbalpha is a critical lithium-sensitive component of the circadian clock. Science 311: 1002-1005.

25. Lopez-Molina L, Conquet F, Dubois-Dauphin M, Schibler U (1997) The DBP gene is expressed according to a circadian rhythm in the suprachiasmatic nucleus and influences circadian behavior. Embo J 16: 6762-6771.

26. Ohno T, Onishi Y, Ishida N (2007) The negative transcription factor E4BP4 is associated with circadian clock protein PERIOD2. Biochem Biophys Res Commun 354: 1010-1015.

27. Etchegaray JP, Yang X, DeBruyne JP, Peters AH, Weaver DR, et al. (2006) The polycomb group protein EZH2 is required for mammalian circadian clock function. J Biol Chem 281: 21209-21215.

28. Cheng HY, Dziema H, Papp J, Mathur DP, Koletar M, et al. (2006) The molecular gatekeeper Dexras1 sculpts the photic responsiveness of the mammalian circadian clock. J Neurosci 26: 12984-12995.

29. Partch CL, Shields KF, Thompson CL, Selby CP, Sancar A (2006) Posttranslational regulation of the mammalian circadian clock by cryptochrome and protein phosphatase 5. Proc Natl Acad Sci U S A 103: 10467-10472.

30. Reischl S, Vanselow K, Westermark PO, Thierfelder N, Maier B, et al. (2007) Beta-TrCP1-mediated degradation of PERIOD2 is essential for circadian dynamics. J Biol Rhythms 22: 375-386.

31. Vielhaber E, Eide E, Rivers A, Gao ZH, Virshup DM (2000) Nuclear entry of the circadian regulator mPER1 is controlled by mammalian casein kinase I epsilon. Mol Cell Biol 20: 4888-4899.

32. Akashi M, Tsuchiya Y, Yoshino T, Nishida E (2002) Control of intracellular dynamics of mammalian period proteins by casein kinase I epsilon (CKIepsilon) and CKIdelta in cultured cells. Mol Cell Biol 22: 1693-1703.

33. Griffin EA, Jr., Staknis D, Weitz CJ (1999) Light-independent role of CRY1 and CRY2 in the mammalian circadian clock. Science 286: 768-771.

34. Oster H, Baeriswyl S, Van Der Horst GT, Albrecht U (2003) Loss of circadian rhythmicity in aging mPer1-/-mCry2-/- mutant mice. Genes Dev 17: 1366-1379.

35. Toh KL, Jones CR, He Y, Eide EJ, Hinz WA, et al. (2001) An hPer2 phosphorylation site mutation in familial advanced sleep phase syndrome. Science 291: 1040-1043.

36. Vanselow K, Vanselow JT, Westermark PO, Reischl S, Maier B, et al. (2006) Differential effects of PER2 phosphorylation: molecular basis for the human familial advanced sleep phase syndrome (FASPS). Genes Dev 20: 2660-2672.

37. Xu Y, Toh KL, Jones CR, Shin JY, Fu YH, et al. (2007) Modeling of a human circadian mutation yields insights into clock regulation by PER2. Cell 128: 59-70.

38. Kume K, Zylka MJ, Sriram S, Shearman LP, Weaver DR, et al. (1999) mCRY1 and mCRY2 are essential components of the negative limb of the circadian clock feedback loop. Cell 98: 193-205.

39. Miyazaki K, Mesaki M, Ishida N (2001) Nuclear entry mechanism of rat PER2 (rPER2): role of rPER2 in nuclear localization of CRY protein. Mol Cell Biol 21: 6651-6659.

40. Xu Y, Padiath QS, Shapiro RE, Jones CR, Wu SC, et al. (2005) Functional consequences of a CKIdelta mutation causing familial advanced sleep phase syndrome. Nature 434: 640-644.

41. Shearman LP, Sriram S, Weaver DR, Maywood ES, Chaves I, et al. (2000) Interacting molecular loops in the mammalian circadian clock. Science 288: 1013-1019.

42. Eide EJ, Kang H, Crapo S, Gallego M, Virshup DM (2005) Casein kinase I in the mammalian circadian clock. Methods Enzymol 393: 408-418.

43. Eide EJ, Vielhaber EL, Hinz WA, Virshup DM (2002) The circadian regulatory proteins BMAL1 and cryptochromes are substrates of casein kinase Iepsilon. J Biol Chem 277: 17248-17254.

44. St-Pierre B, Flock G, Zacksenhaus E, Egan SE (2002) Stra13 homodimers repress transcription through class B E-box elements. J Biol Chem 277: 46544-46551.

45. Hogenesch JB, Gu YZ, Jain S, Bradfield CA (1998) The basic-helix-loop-helix-PAS orphan MOP3 forms transcriptionally active complexes with circadian and hypoxia factors. Proc Natl Acad Sci U S A 95: 5474-5479.

46. McNamara P, Seo SB, Rudic RD, Sehgal A, Chakravarti D, et al. (2001) Regulation of CLOCK and MOP4 by nuclear hormone receptors in the vasculature: a humoral mechanism to reset a peripheral clock. Cell 105: 877-889.

47. Gietzen KF, Virshup DM (1999) Identification of inhibitory autophosphorylation sites in casein kinase I epsilon. J Biol Chem 274: 32063-32070.

48. Kim MS, Lee YT, Kim JM, Cha JY, Bae YS (1998) Characterization of protein interaction among subunits of protein kinase CKII in vivo and in vitro. Mol Cells 8: 43-48.

49. Lehner B, Semple JI, Brown SE, Counsell D, Campbell RD, et al. (2004) Analysis of a high-throughput yeast two-hybrid system and its use to predict the function of intracellular proteins encoded within the human MHC class III region. Genomics 83: 153-167.

50. Marin O, Meggio F, Sarno S, Pinna LA (1997) Physical dissection of the structural elements responsible for regulatory properties and intersubunit interactions of protein kinase CK2 beta-subunit. Biochemistry 36: 7192-7198.

51. Ahn BH, Kim TH, Bae YS (2001) Mapping of the interaction domain of the protein kinase CKII beta subunit with target proteins. Mol Cells 12: 158-163.

52. Litchfield DW, Lozeman FJ, Cicirelli MF, Harrylock M, Ericsson LH, et al. (1991) Phosphorylation of the beta subunit of casein kinase II in human A431 cells. Identification of the autophosphorylation site and a site phosphorylated by p34cdc2. J Biol Chem 266: 20380-20389.

53. Sablina AA, Chen W, Arroyo JD, Corral L, Hector M, et al. (2007) The tumor suppressor PP2A Abeta regulates the RalA GTPase. Cell 129: 969-982.

54. Kitajima TS, Sakuno T, Ishiguro K, Iemura S, Natsume T, et al. (2006) Shugoshin collaborates with protein phosphatase 2A to protect cohesin. Nature 441: 46-52.

55. Shern JF, Sharer JD, Pallas DC, Bartolini F, Cowan NJ, et al. (2003) Cytosolic Arl2 is complexed with cofactor D and protein phosphatase 2A. J Biol Chem 278: 40829-40836.

56. McCright B, Rivers AM, Audlin S, Virshup DM (1996) The B56 family of protein phosphatase 2A (PP2A) regulatory subunits encodes differentiation-induced phosphoproteins that target PP2A to both nucleus and cytoplasm. J Biol Chem 271: 22081-22089.

57. Rual JF, Venkatesan K, Hao T, Hirozane-Kishikawa T, Dricot A, et al. (2005) Towards a proteome-scale map of the human protein-protein interaction network. Nature 437: 1173-1178.

58. Tamaru T, Hirayama J, Isojima Y, Nagai K, Norioka S, et al. (2009) CK2alpha phosphorylates BMAL1 to regulate the mammalian clock. Nat Struct Mol Biol 16: 446-448.

59. Langmesser S, Tallone T, Bordon A, Rusconi S, Albrecht U (2008) Interaction of circadian clock proteins PER2 and CRY with BMAL1 and CLOCK. BMC Mol Biol 9: 41.

60. Chaurasia G, Futschik M (2012) The integration and annotation of the human interactome in the UniHI Database. Methods Mol Biol 812: 175-188.

61. Zylka MJ, Shearman LP, Levine JD, Jin X, Weaver DR, et al. (1998) Molecular analysis of mammalian timeless. Neuron 21: 1115-1122.

62. Gallego M, Kang H, Virshup DM (2006) Protein phosphatase 1 regulates the stability of the circadian protein PER2. Biochem J 399: 169-175.

63. Iitaka C, Miyazaki K, Akaike T, Ishida N (2005) A role for glycogen synthase kinase-3beta in the mammalian circadian clock. J Biol Chem 280: 29397-29402.

64. Ohno T, Onishi Y, Ishida N (2007) A novel E4BP4 element drives circadian expression of mPeriod2. Nucleic Acids Res 35: 648-655.

65. Harada Y, Sakai M, Kurabayashi N, Hirota T, Fukada Y (2005) Ser-557-phosphorylated mCRY2 is degraded upon synergistic phosphorylation by glycogen synthase kinase-3 beta. J Biol Chem 280: 31714-31721.

66. Heriche JK, Lebrin F, Rabilloud T, Leroy D, Chambaz EM, et al. (1997) Regulation of protein phosphatase 2A by direct interaction with casein kinase 2alpha. Science 276: 952-955.

67. Zhou J, Pham HT, Ruediger R, Walter G (2003) Characterization of the Aalpha and Abeta subunit isoforms of protein phosphatase 2A: differences in expression, subunit interaction, and evolution. Biochem J 369: 387-398.

68. Lubert EJ, Hong Y, Sarge KD (2001) Interaction between protein phosphatase 5 and the A subunit of protein phosphatase 2A: evidence for a heterotrimeric form of protein phosphatase 5. J Biol Chem 276: 38582-38587.

69. Wang J, Yin L, Lazar MA (2006) The orphan nuclear receptor Rev-erb alpha regulates circadian expression of plasminogen activator inhibitor type 1. J Biol Chem 281: 33842-33848.

70. Schmutz I, Ripperger JA, Baeriswyl-Aebischer S, Albrecht U (2009) The mammalian clock component PERIOD2 coordinates circadian output by interaction with nuclear receptors. Genes Dev 24: 345-357.

71. Suzuki H, Chiba T, Suzuki T, Fujita T, Ikenoue T, et al. (2000) Homodimer of two F-box proteins betaTrCP1 or betaTrCP2 binds to IkappaBalpha for signal-dependent ubiquitination. J Biol Chem 275: 2877-2884.
